# Ultrastrong *Staphylococcus aureus* adhesion to human skin: Calcium as a key regulator of noncovalent interactions

**DOI:** 10.1126/sciadv.adu7457

**Published:** 2025-09-03

**Authors:** Constance Chantraine, Priscila S. F. C. Gomes, Marion Mathelié-Guinlet, Diego E. B. Gomes, Zhiyong Zheng, Julianne Clowry, Mary B. Turley, Alan D. Irvine, Joan A. Geoghegan, Rafael C. Bernardi, Yves F. Dufrêne

**Affiliations:** ^1^ Louvain Institute of Biomolecular Science and Technology, UCLouvain, Louvain-la-Neuve, Belgium.; ^2^ Department of Physics, Auburn University, Auburn, AL, USA.; ^3^ Institute of Chemistry and Biology of Membranes and Nano-objects, UMR 5248, CNRS, Bourdeaux INP, University of Bordeaux, Pessac, France.; ^4^ Clinical Medicine, Trinity College Dublin, Dublin, Ireland.; ^5^ Department of Microbes, Infection and Microbiomes, University of Birmingham, Birmingham, UK.; ^6^ Institute of Microbiology and Infection, University of Birmingham, Birmingham, UK.; ^7^ Department of Microbiology, School of Genetics and Microbiology, Trinity College Dublin, Dublin, Ireland.

## Abstract

Calcium is a critical regulator of *Staphylococcus aureus* skin adhesion, stabilizing one of the strongest noncovalent biomolecular interactions ever recorded. Using in vitro and in silico single-molecule force spectroscopy, we demonstrate that calcium ions (Ca^2+^) are essential for the ultrastrong binding between the serine-aspartate repeat protein D (SdrD) adhesin and the human skin protein desmoglein-1 (DSG-1), withstanding forces exceeding 2 nanonewtons. Ca^2+^ ions stabilize both the SdrD complex and the mechanically robust SdrD B-domains, which exhibit unprecedented folding strength. In the context of atopic dermatitis (AD), disrupted calcium gradients amplify SdrD interactions, which could potentially intensify *S. aureus* virulence. Furthermore, abnormal DSG-1 distribution on AD-affected skin enhances bacterial adhesion. These findings provide crucial insights into the calcium-dependent regulation of bacterial adhesion and folding, suggesting possible therapeutic targets to combat *S. aureus* infections.

## Teaser

Calcium ions stabilize one of the strongest known noncovalent biological interactions.

## INTRODUCTION

The Gram-positive bacterium *Staphylococcus aureus* is a commensal organism found in ~20% of healthy adults (*1*–*3*). It is also an opportunistic pathogen capable of causing a broad spectrum of infections, ranging from superficial skin lesions to severe, life-threatening conditions such as endocarditis and septicemia. *S. aureus* is also known to exacerbate symptoms of atopic dermatitis (AD; eczema) (*4*–*7*), and its presence has been demonstrated to correlate with disease severity (*8*–*11*). The rising incidence of *S. aureus* infections is driven by increased colonization rates, a higher prevalence of immunosuppressive conditions, the widespread use of surgical implants, and the expansion of antibiotic resistance (*12*, *13*). The emergence of methicillin-resistant *S. aureus* strains has further exacerbated treatment challenges by expanding resistance to multiple antibiotics (*14*, *15*). Despite extensive research efforts, no vaccine against *S. aureus* has been approved to date (*15*, *16*).

Skin colonization by *S. aureus* begins with its attachment to the stratum corneum (SC), the outermost layer of the epidermis (*17*). The SC consists of corneocytes, which are flattened dead cells filled with keratin and surrounded by a cornified envelope, in contrast to the living keratinocytes found in deeper layers. Corneocytes are connected by corneodesmosomes, primarily composed of desmoglein-1 (DSG-1), corneodesmosin (CDSN), and desmocollin-1. These proteins are localized only at the periphery of the cells (*18*), suggesting that their degradation at the corneocyte center could provide space for the stable maintenance of intercellular lipids (*19*). DSG-1 is expressed on the surface of both corneocytes and keratinocytes. This cadherin-type cell adhesion protein has a calcium-dependent extracellular (EC) region with four cadherin repeats, an anchor domain, a transmembrane region, and a cytoplasmic domain (see fig. S1) (*20*). *S. aureus* exfoliative toxins cleave the homophilic interaction site of DSG-1, causing cell detachment (*21*) and compromising the SC barrier function (*19*). Mutations in DSG-1 are linked to dermatitis, multiple allergies, and metabolic wasting (*22*), highlighting DSG-1’s essential role in maintaining skin integrity and its involvement in disease.

*S. aureus* adheres to corneocytes using cell wall–anchored surface proteins, including fibronectin-binding proteins A (FnBPA) and B (FnBPB), clumping factor B (ClfB), and iron-regulated surface determinant A (IsdA) (*6*, *23*, *24*). These adhesins form mechanostable bonds (*25*, *26*) with target ligands such as keratin, loricrin, and CDSN. ClfB mediates *S. aureus* adhesion to corneocytes from patients with AD by binding to loricrin and cytokeratin via the dock, lock, and latch (DLL) mechanism (*27*, *28*). Both FnBPB and ClfB interact with the N-terminal region of CDSN on AD corneocytes. An important challenge is to identify other adhesins that may play roles in the colonization of skin, their target ligands, and their binding mechanisms. The serine-aspartate repeat protein D (SdrD) was identified as a potential partner for DSG-1, which has been shown to promote *S. aureus* adhesion to human keratinocytes (*29*) and desquamated nasal epithelial cells (*30*), although its mechanisms remain unknown.

**Fig. 1.**
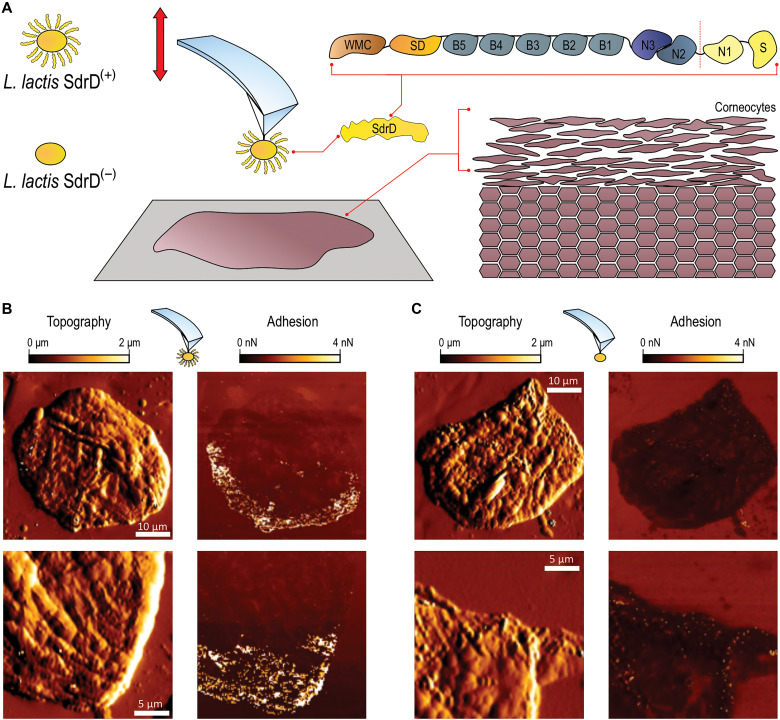
SdrD mediates bacterial adherence to healthy corneocytes. (**A**) Schematic illustration of SCFS experiments where we mapped the molecular interactions between single *L. lactis* SdrD^(+)^ and *L. lactis* SdrD^(−)^ bacterial probes and corneocytes. SdrD different functional domains are described on an insert: WMC (Wall- and membrane-spanning domains); SD (serine-aspartate repeats); B-repeated regions; the A region comprising N1, N2, and N3 domains; and the S (signal peptide), removed posttranslationally. (**B** and **C**) Representative topographic images of the structure and adhesion of corneocytes recorded with *L. lactis* SdrD^(+)^ and *L. lactis* SdrD^(−)^, respectively. Adhesion featuring numerous strong rupture events is shown as bright pixels. One of the experiments is represented, over an *n* = 3 cells.

SdrD is part of the Sdr family of *S. aureus* surface proteins, characterized by an N-terminal signal peptide, followed by an A region composed of N1, N2, and N3 subdomains, variable numbers of B-repeats (110 to 113 residues), a Ser-Asp repeat (R) domain, and an Leu-Pro-X-Thr-Gly (LPXTG) cell wall–anchoring motif (see Fig. 1A and fig. S1) (*31*). The B-repeats of SdrD form a rigid rod-like structure, proposed to function as shock absorbers that project the A-domain away from the cell surface, with Ca^2+^ required for proper folding and structural integrity (see fig. S1) (*25*, *32*, *33*).

The remarkable architecture of *S. aureus* adhesins allows them to form mechanical bonds that are not only highly specific but also extremely resilient under force (*34*). Such mechanostable interactions are crucial for pathogen survival in dynamic environments like the skin or bloodstream, where shear forces constantly challenge adhesion. Several staphylococcal adhesins, including ClfA, ClfB, and the serine-aspartate repeat protein G (SdrG), form some of the strongest noncovalent protein-protein bonds known to date, with rupture forces exceeding 2 nN (*35*). These interactions are characteristic examples of catch bonds, whose lifetimes increase under mechanical load, thereby reinforcing bacterial attachment during colonization (*36*). Mechanostable bonds are not limited to bacterial systems. They are also prevalent in viral infections (*37*–*40*), where force-dependent interactions can regulate viral entry and immune evasion, and are increasingly recognized in the context of genetic diseases involving mechanically sensitive protein complexes (*41*, *42*). Beyond their roles in pathogenesis, these force-enhanced interactions have been widely adopted in biotechnology. Applications range from the well-known streptavidin:biotin pair (*43*, *44*), valued for its extreme stability, to engineered therapeutic complexes such as PD-L1:affibody (*45*), which rely on tethering-specific mechanical resilience for improved performance.

In this study, we used single-cell force spectroscopy (SCFS) to investigate the binding of SdrD to healthy and AD corneocytes, focusing on the roles of DSG-1 and calcium in the adhesion mechanism. Our findings reveal that SdrD forms exceptionally mechanostable bonds with corneocytes, particularly DSG-1, enduring forces greater than 2 nN. Notably, the adhesion frequency was clearly higher in AD corneocytes compared to healthy cells. By combining in vitro and in silico single-molecule force spectroscopy (SMFS), we identified the SdrD binding site and demonstrated that calcium stabilizes the complex by reinforcing the adjacent cadherin domains. The extraordinary hyperstability of the SdrD complex, which represents the strongest molecular complex known to date, is explained by a highly connected network of interactions, where the E-cadherin domain [extracellular domain 4 (EC4)] bridges key interface connections, further rigidifying the complex. These findings suggest that the DLL mechanism in this system is enhanced by the interplay between DSG-1 cadherins and the A-domain of SdrD.

## RESULTS

### Characterizing SdrD-mediated adhesion to corneocytes

To test whether SdrD promotes bacterial adhesion to corneocytes, we first mapped the molecular interactions between single bacterial probes and corneocytes from the SC of healthy subjects using SCFS in the quantitative imaging (QI) mode (*46*–*48*). In this method, a *Lactococcus lactis* bacterium, as a model system, is bound to the cantilever of an atomic force microscope, which is then moved over a surface containing the corneocytes. In our study, *L. lactis* bacteria expressing SdrD on their surface, SdrD^(+)^, and bacteria lacking this protein, SdrD^(−)^, were used, as shown in Fig. 1A. Adherence assays confirmed surface expression of SdrD in *L. lactis* SdrD^(+)^ (fig. S2). Representative correlated images of the topography and adhesion of corneocytes recorded with *L. lactis* SdrD^(+)^ bacterial probes are shown in Fig. 1B. Topographic images revealed fine structural details of the skin cell surfaces despite the large probe size. Notably, adhesion images featured numerous strong adhesion events (bright pixels), primarily localized on the edges of the corneocytes. These interactions were abolished when using *L. lactis* SdrD^(−)^ probes (Fig. 1C), indicating that they are exclusively mediated by SdrD.

**Fig. 2.**
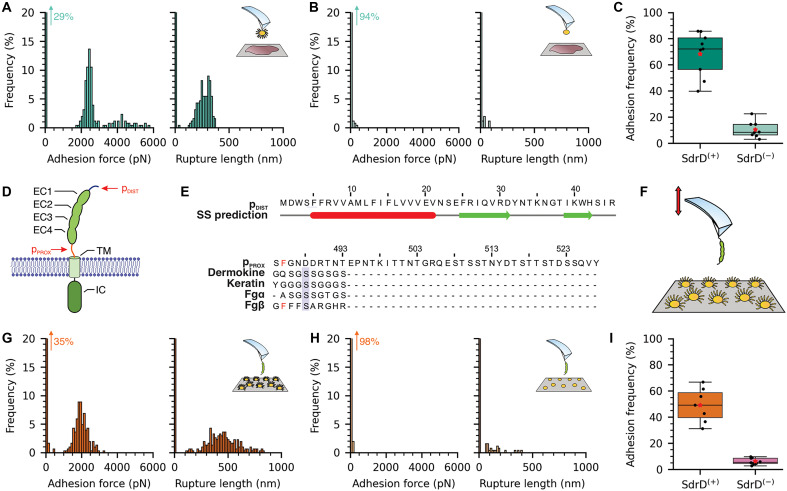
DSG-1 is the target ligand of SdrD. (**A**) *L. lactis* SdrD^(+)^–dependent adhesion rupture forces were extremely strong, with a sharp distribution centered at 2336 pN. Rupture lengths are around 265 nm, consistent with the length of the adhesin. A representative histogram is shown (*n* = 256). (**B**) No adhesion rupture events are observed for *L. lactis* SdrD^(−)^. A representative histogram is shown (*n* = 256). (**C**) Adhesion frequencies estimated over the SCFS experiments for *L. lactis* SdrD^(+)^ and *L. lactis* SdrD^(−)^. (**D**) Schematic view for the DSG-1 protein, highlighting its four EC domains, the transmembrane (TM) region and the intracellular (IC) domain. Red arrows represent two unfolded regions that could be the potential binding sites for SdrD. (**E**) Bioinformatics analysis on two peptides located near EC1 and EC4, here called p_DIST_ and p_PROX_, respectively. p_DIST_ sequence has propensity to form β strands, while p_PROX_ sequence does not; nevertheless, alignment with other peptides cocrystallized with ClfB, dermokine (PDB ID: 4F20), keratin (PDB ID: 4F1Z), and fibrinopeptide-α (PDB ID: 4F27), reveals a potential region near the transmembrane domain of DSG-1. In addition, we observe a conservation of a Phe residue present at fibrinogen-β (Fgβ) (PDB ID: 1R17) that plays a key role for SdrG:Fgβ adhesion. (**F**) Schematic representation for the SMFS experiment to investigate the binding of SdrD to DSG-1 by recording the force curves between *L. lactis* SdrD^(+)^ and *L. lactis* SdrD^(−)^ and atomic force microscopy (AFM) tips functionalized with DSG-1. (**G**) Similar to the values obtained with the full corneocytes, *L. lactis* SdrD^(+)^ adhesion rupture forces were very strong, around 2076 pN. A representative histogram is shown (*n* = 304). (**H**) No adhesion events were observed for the construct *L. lactis* SdrD^(−)^. A representative histogram is shown (*n* = 256). (**I**) Adhesion frequencies estimated over the SMFS experiments for *L. lactis* SdrD^(+)^ and *L. lactis* SdrD^(−)^.

To further characterize the interaction between SdrD and corneocytes, SCFS in the force-volume (FV) imaging mode was used. Figure 2 (A and C) and fig. S3 show a remarkable mechanostable adhesion between *L. lactis* SdrD^(+)^ and corneocytes, with a high adhesion frequency, while *L. lactis* SdrD^(−)^ did not exhibit any mechanostable events (Fig. 2, B and C, and fig. S4). For *L. lactis* SdrD^(+)^, ~70% of the force curves featured adhesive events, whereas these events were largely absent in *L. lactis* SdrD^(−)^ (Fig. 2C), underscoring the role of SdrD in the observed interactions. Additionally, SdrD-dependent adhesion forces were notably strong, with a sharp distribution centered at 2336 ± 263 pN (means ± SD), estimated from 2721 adhesion force profiles from nine different bacterium-corneocyte pairs (Fig. 2A and fig. S3). We propose that the 2.3-nN forces correspond to the rupture of single SdrD-ligand complexes, as similar narrow distributions of high forces were consistently observed in all cell pairs. Additionally, a less frequent population of unbinding forces peaked at 4135 ± 340 pN was observed, likely due to the rupture of two bonds in parallel (Fig. 2A and fig. S3). These bonds always ruptured at the same distance, 272 ± 62 nm, indicating parallel rather than serial bond ruptures. The binding strength of a single SdrD:corneocyte pair is comparable to that of the prototypical DLL interaction between SdrG and fibrinogen-β (Fgβ) (*35*), suggesting that SdrD-ligand binding may proceed through a similar DLL mechanism.

Among the constituents of corneodesmosomes, SdrD has been shown to be able to bind DSG-1 (*29*). Binding events were mostly distributed on the periphery of healthy corneodesmosomes, which have surfaces coated with DSG-1 proteins. Computational modeling and bioinformatics analyses were used to characterize the interaction between DSG-1 and SdrD. The first step was to predict the tridimensional structures of the outer membrane domains of DSG-1, which comprise four EC domains (Fig. 2D and fig. S1). Using this strategy, we identified two loop regions on DSG-1 as candidates for binding at the N2N3 interface of the SdrD A-domain. Both regions were primarily unstructured. Region 1, distal from the cell membrane at the N-terminal region of DSG-1, was predicted by bioinformatic analysis to form a β sheet with SdrD, resembling other adhesin structures (*49*–*53*). Region 2, proximal to the membrane, between the E-cadherin EC4 and the transmembrane region, did not show a stable secondary structure formation with SdrD (see Fig. 2E). Examining the crystal structure of the unbound (apo) form of SdrD [Protein Data Bank (PDB) ID: 4JE0] showed a high structural and sequence similarity in the peptide-binding area to that of ClfB (PDB ID: 4F27). We then aligned the protein sequence of Region 2 with the possible targets of ClfB found in the PDB (*53*). Our analysis also considered the Fgβ complex with SdrG (PDB ID: 1R17) (*49*). Notably, one of the bulky phenylalanine (Phe) residues from Fgβ aligns with Phe^486^ on DSG-1, a residue crucial for maintaining the integrity of the SdrG:Fgβ complex (*35*). Within these two potential target regions, we selected two peptide sequences, here named p_DIST_ and p_PROX_ (Fig. 2E).

To confirm DSG-1 as the binding target of SdrD, we conducted SMFS using atomic force microscopy (AFM) tips modified with purified, full-length DSG-1 (Fig. 2F) to examine the binding affinity and mechanostability of the SdrD on living bacteria. The SMFS experiments, shown in Fig. 2G and fig. S5, revealed strong adhesion forces for *L. lactis* SdrD^(+)^, similar to those obtained with full corneocytes, at 2076 ± 248 pN, from 1229 adhesion force profiles (seven different cells). In contrast, *L. lactis* SdrD^(−)^ showed no mechanostable events (Fig. 2H and fig. S6). These results confirm DSG-1 as the SdrD target, which is also demonstrated by a drop in binding probability from 49% for SdrD^(+)^ to 6% for SdrD^(−)^ (Fig. 2I). Again, the high-force populations were comparable to the prototypical DLL interaction between SdrG and Fgβ (*35*, *49*, *54*, *55*). Unlike SCFS experiments, we did not observe higher forces associated with multiple parallel interactions, consistent with the lower density of interacting molecules on SMFS tips compared to SCFS bacterial probes. The rupture lengths were larger with DSG-1 than with corneocytes, averaging 431 ± 104 nm (Fig. 2, A and G, and fig. S5). This difference can be attributed to the transmembrane nature of DSG-1: While SMFS examines the full-length protein (1049 amino acids), SCFS focuses solely on its EC region (548 amino acids). The rupture length profile suggests that p_PROX_ is the likely binding target. The similarity in strong binding forces measured for SdrD binding to DSG-1 and to healthy corneocytes strongly suggests that the binding partners interact in situ. This is further supported by the structure of DSG-1, whose interacting region is located in the A-domain and likely involves a DLL mechanism, as revealed on corneocytes.

**Fig. 3.**
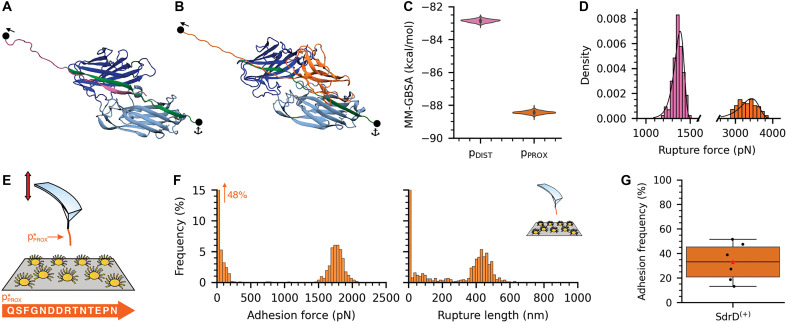
SdrD binding site is predicted in silico and validated in vitro. (**A** and **B**) Three-dimensional (3D) structures for SdrD:DSG-1 complex with p_DIST_ or p_PROX_ at the interface, respectively. SdrD A-domain, represented by N2 and N3 domains are colored in dark and light blue, respectively, while p_DIST_ is colored in pink and p_PROX_ is colored in light orange. Anchor and pulling points used in the steered molecular dynamics (SMD) simulations are also indicated. Due to the physical proximity between p_PROX_ and EC4, the latter was included on SdrD:DSG-1 model, colored in bright orange. (**C**) Free energy of binding between SdrD and p_DIST_ or p_PROX_, estimated from in-equilibrium MD simulations. (**D**) Adhesion rupture forces observed during SMD simulations for SdrD:p_DIST_ and SdrD:P_PROX_ complexes. Values observed for SdrD:p_PROX_ are clearly higher and pinpoint p_PROX_ as the probable binding site for SdrD. *n* = 128 and *n* = 126 for p_DIST_ and p_PROX_, respectively. Pulling speed, 5.0 × 10^−4^ nm/ps. (**E**) Schematic view of the *L. lactis* SdrD^(+)^:$p_{\text{PROX}}^{*}$ SMFS experiment; the sequence of $p_{\text{PROX}}^{*}$ peptide, based on p_PROX_, is shown. (**F**) Adhesion rupture forces between *L. lactis* SdrD^(+)^ and $p_{\text{PROX}}^{*}$ . Strong rupture forces were detected around 1808 pN with rupture lengths around 400 nm (*n* = 1024). Pulling speed, 1 μm s^−1^. (**G**) Adhesion frequencies estimated over the SMFS experiments for *L. lactis* SdrD^(+)^:$p_{\text{PROX}}^{*}$ . The boxplot shows data from *n* = 6 cells; red star indicates the mean value. Events with no adhesion were not considered on the boxplot.

The SMFS experiments demonstrate that DSG-1 is the target of SdrD. While these experiments provided strong indications, particularly through the rupture length profiles, they could not definitively confirm whether p_PROX_ or p_DIST_ of DSG-1 is the specific binding region. To elucidate which region SdrD binds to, we conducted extensive computational experiments using an in silico SMFS approach (*56*). First, using MODELLER (*57*), we built all-atom three-dimensional (3D) models of the DSG-1 peptides p_DIST_ and p_PROX_ in complex with SdrD based on the solved apo structure of SdrD (*33*) and the crystallographic structure of SdrG complexed with Fgβ (Fig. 3, A and B) (*49*). Specific to p_PROX_, we incorporated the fourth EC E-cadherin domain (EC4) of DSG-1 into the model to assess its influence on the mechanostability of the complex, as this domain was found to be near the potential binding region (Fig. 3B). Using graphic processing units (GPU)-accelerated NAMD3 (*58*) through its QwikMD interface (*59*), we performed classical all-atom molecular dynamics (MD) simulations on explicitly solvated SdrD:DSG-1 models. The binding free energy of p_DIST_ and p_PROX_ to SdrD was estimated using the molecular mechanics with generalized Born (GB) and surface area solvation (MM-GBSA) (Fig. 3C) (*60*, *61*). The results show that p_PROX_ has a stronger binding affinity than p_DIST_, with a difference of 5 kcal/mol.

We then performed all-atom steered MD (aa-SMD) simulations to determine the rupture forces for the SdrD complexes with both p_DIST_ and p_PROX_. The distribution of these forces, following the Bell-Evans (BE) fit, is presented in Fig. 3D. SdrD:p_DIST_ rupture forces showed a sharp distribution centered at 1360 ± 56 pN, while SdrD:p_PROX_ rupture forces displayed a broader distribution centered at 3321 ± 56 pN. These data indicate that the SdrD:p_PROX_ complex exhibits greater mechanostability compared to the SdrD:p_DIST_ complex. The rupture forces for SdrD:p_DIST_ were low for adhesins (*34*, *35*, *62*). It has been previously hypothesized, through a combination of in silico and in vitro SMFS, that any peptide binding an adhesin via a DLL mechanism would exhibit nanonewton mechanostability at AFM-level force loading rates (*35*). However, due to limitations in computational methods, in silico force loading rates are much higher, resulting in higher rupture forces (*35*). Therefore, the ~1.4-nN rupture forces measured by in silico SMFS suggest that rupture forces would be a fraction of nanonewton if tested with in vitro SMFS experiments. This result suggests that only p_PROX_ could account for the high-force regimes observed in the AFM experiments presented in Fig. 2G and fig. S5. Additionally, the relatively low rupture force observed for SdrD:p_DIST_ may indicate a modified version of the traditional DLL mechanism. A visual inspection of our simulations revealed that the EC4 domain likely contributes to the high mechanostability of the SdrD:p_PROX_ interface. Also, the binding configuration of p_PROX_ and the high rupture forces observed are consistent with those seen for SdrG (*36*, *55*, *63*).

To further validate our in silico predictions, we performed SMFS experiments using a peptide construct derived from p_PROX_, consisting of a shorter sequence encompassing only the main predicted binding interface. This minimal construct is hereafter referred to as $p_{\text{PROX}}^{*}$ (Fig. 3E). Consistent with the results shown in Fig. 2G for the full-length DSG-1 construct, binding of $p_{\text{PROX}}^{*}$ to SdrD exhibits nanonewton-range mechanostability, with rupture forces displaying a sharp distribution centered at 1808 ± 196 pN and adhesion frequencies around 33% (Fig. 3, F and G, and fig. S7). The rupture lengths are ~460 ± 122 nm, closely matching those observed in the full-protein experiments. These results confirm that p_PROX_ contains the correct binding site within DSG-1 and that a single interaction with SdrD is highly force resilient, remaining stable under nanonewton-scale loads.

### Mechanostability and sequential domain unfolding of the SdrD complex

**Fig. 4.**
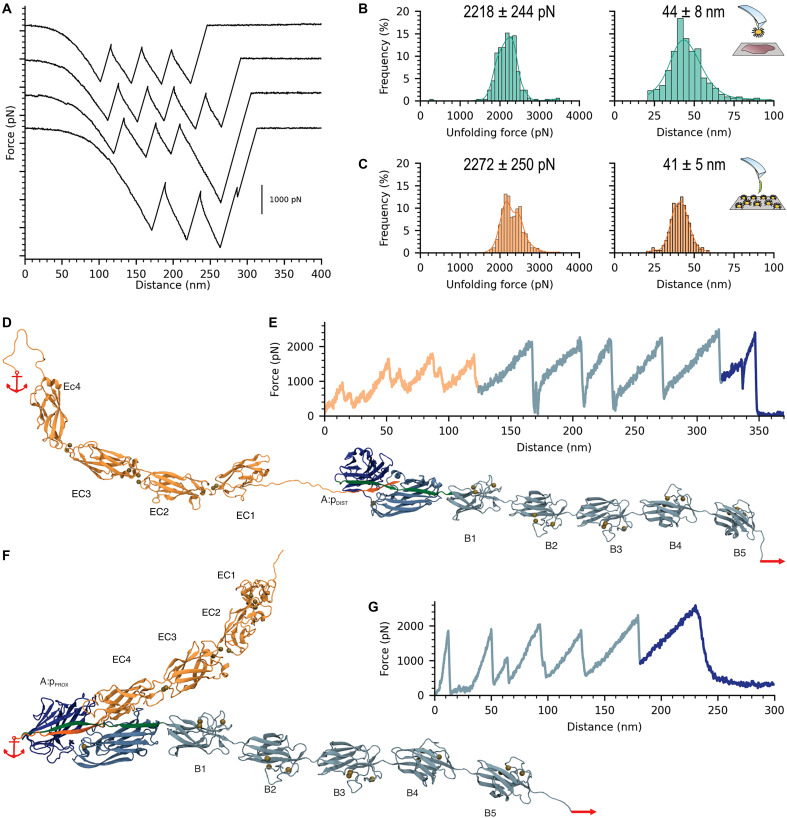
Adhesin B-domains unfolding forces are equally strong and follow a consistent pattern. (**A**) Canonical sawtooth pattern detected on SCFS experiments for *L. lactis* SdrD^(+)^ binding to corneocytes. (**B** and **C**) Unfolding forces observed for SCFS of *L. lactis* SdrD^(+)^ binding to corneocytes and SMFS of *L. lactis* SdrD^(+)^ binding to DSG-1, respectively. Peak-to-peak distances match the expected length for fully extended B-domains [*n* = 337 and *n* = 353 for (B) and (C), respectively]. (**D** and **F**) 3D structure of the full-length model for SdrD:DSG-1 where p_DIST_ or p_PROX_ is at the complex interface, respectively. The different domains are indicated in the image and anchor and pulling points used for SMD simulations are also displayed. Color code for SdrD N2 and N3 domains and DSG-1 is the same as in Fig. 2. B-domains are colored in teal. (**E**) Representative force-versus-extension curves for the full-length SdrD:DSG-1^pDIST^ model. Curves are colored by protein domain. We observed distinct unfolding patterns for the DSG-1 EC domains, followed by SdrD five B-domains and, lastly, the rupture of the interaction between SdrD N2-N3 and p_DIST_. (**G**) Representative force-versus-extension curves for the full-length SdrD:DSG-1^pPROX^ model. Curves are colored by protein domain. We observed distinct unfolding patterns for SdrD five B-domains and, lastly, the rupture of the interaction between SdrD N2-N3 and p_PROX_, consistent with observed experimentally, corroborating that p_PROX_ is the probable binding site for SdrD.

The SMD results showing high resilience to mechanical load for p_PROX_ strongly suggest that it is the binding site for the SdrD A-domain. The SMFS experiments with $p_{\text{PROX}}^{*}$ confirmed these finds. However, looking only at rupture events would be a limited use of the experimental SMFS traces. SdrD^(+)^:corneocyte experiments exhibited a large number, 91% (2339 of the 2570 curves), of adhesive curves featuring sawtooth patterns with multiple (mostly 2, 3, or 4) equally spaced force peaks preceding the final rupture peak (Fig. 4A). The canonical sawtooth pattern was also detected with SdrD^(+)^:DSG-1, with unfolding forces and peak-to-peak distances similar to those observed in corneocytes (Fig. 4, B and C). Same can be said about the single-molecule experiments with $p_{\text{PROX}}^{*}$ (fig. S8). These profiles reflect the force-induced unfolding of protein secondary structures (α helices and β sheets) (*64*) and have been reported for the multirepeat staphylococcal adhesins SasG (*65*) and Aap (*66*).

The SdrD^(+)^:corneocyte signatures featured unfolding forces of 2213 ± 267 pN in 2339 adhesion force profiles (nine cell pairs) and a mean peak-to-peak distance of 43 ± 7 nm (Fig. 4B), matching the length expected for fully extended B-domain immunoglobulin-like folds (110 amino acids × 0.36 nm per residue, 4 nm for the folded domain). A similar behavior was observed for SdrD^(+)^:DSG-1 (Fig. 4C). Consistent with SMFS experiments on purified SdrG B1 and B2 domains (*67*), our data collected directly on physiologically relevant bacterial-corneocyte systems demonstrate that the force required to unfold individual SdrD B-domains is extremely high, representing the strongest protein fold reported thus far.

The sawtooth behavior of the force traces presented in Fig. 4A can also be used as a signature of the binding complex. To investigate the role of B-domains in complex stability and reproduce the adhesive sawtooth patterns observed experimentally, we generated tridimensional models for the full-length SdrD:DSG-1 complexes using a combination of AlphaFold2 (*68*), to generate the individual DSG-1 EC domains and SdrD B-domains, and MODELLER, to combine it with SdrD A-domain. These models included the five B-domains of SdrD and the four EC domains of DSG-1, considering both binding sites where either p_DIST_ or p_PROX_ is at the SdrD:DSG-1 interface (Fig. 4, D and F). The systems were built with all-atoms and solvated in an explicit water box for geometry optimization and energy equilibration. Due to the complexity and size of the model structures, the production SMD runs were performed at the coarse-grained (CG) level using Martini 3 (*69*) implemented in GROMACS (*70*). The pulling simulations were performed by holding on the free C-terminal end of DSG-1 and pulling on the C-terminal end of SdrD. Exemplary traces show multiple peaks in a sawtooth pattern as observed experimentally (Fig. 4, E and G). However, simulations with p_DIST_ as the binding position exhibited extra, lower-force peaks, characteristic of the DSG-1 EC domains unfolding (Fig. 4E). Additionally, the system length exceeded 340 nm, which is too long compared to the experimental traces.

It is important to note that, due to the inherent resolution limits of CG models, particularly their simplified treatment of side-chain interactions and electrostatics, rupture force differences between the two binding modes (p_PROX_ versus p_DIST_) are not well resolved in the CG force-extension traces. This is an expected limitation, as CG simulations are designed to capture global mechanical responses rather than fine interaction details, which are more accurately represented in aa-SMD simulations and experimental SMFS. As a result, the rupture forces observed in dark blue in Fig. 4 (E and G) appear similar for both complexes, in contrast to the distinct rupture forces reported in Fig. 3D.

For the SdrD:DSG-1^pPROX^ model, between three and five main peaks were observed before the main rupture event, depending on the simulation replica. These peaks corresponded to the unfolding of the B-domains and have an unfolding force similar to the rupture force of the A-domain. The force trace profiles obtained for the SdrD:DSG-1^pPROX^ complex (Fig. 4G) align with the experimentally observed sawtooth profile, lacking low rupture peaks that could correspond to EC DSG-1 cadherin domains. Instead, we reproduced the five unfolding force peaks preceding the rupture event.

Visual inspection of the MD trajectories provided insights into the sequential rupture of the domains within the complex. The B-domains underwent rupture in a seemingly stochastic order, followed by dissociation at the A-domain interface. The forces required to induce rupture were comparable across all B-domains, with the A-domain exhibiting marginally elevated forces (Fig. 4G). Based on the force profiles, comparison with experimental data, and these findings, we inferred that p_PROX_ is the binding partner for SdrD. Additionally, these results confirm that, as previously proposed (*67*, *71*), the B-domains function as shock absorbers, enhancing the “survivability” of the A-domain under high force loads.

### Loading rate–dependent adhesion and mechanostability of the SdrD complex

**Fig. 5.**
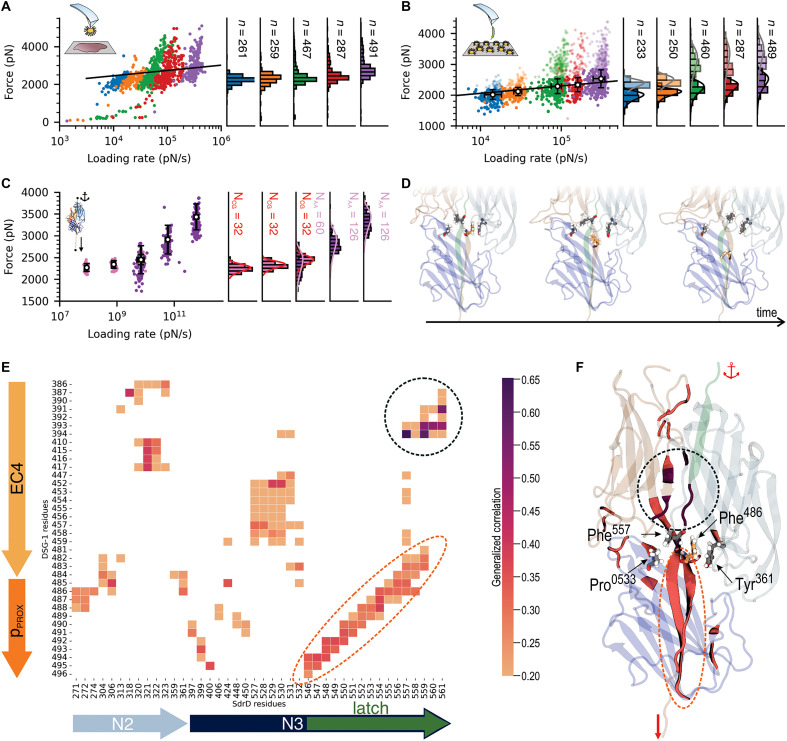
Bond strength between SdrD and DSG-1 increases as mechanical force is applied. Force-dependent activation is evaluated using DFS in vitro and in silico. (**A**) Force versus loading rate for SCFS *L. lactis* SdrD^(+)^ experiments. The 2-nN forces were described by a BE model, with a kinetic off rate (*k*_off_) of 8.8 × 10^−8^ s^−1^ and a transition energetic barrier at (*x*β) of 3.40 × 10^−2^ nm. (**B**) Force versus loading rate for SMFS *L. lactis* SdrD^(+)^ experiments. Adhesion forces were described by a BE model with a *k*_off_ of 1.4 × 10^−7^ s^−1^ and a transition energetic barrier at (*x*β) of 3.96 × 10^−2^ nm, matching those on corneocytes. Only adhesion forces of >1000 pN were considered. (**C**) Force versus loading rate for SMD simulations of SdrD:DSG-1^pPROX^. As for the experiments, at high loading rates, we observe a sharp shift in force that reflects the activation of the complex by mechanical stress. (**D**) SMD snapshots showing the aromatic π-stacking interactions at the SdrD:DSG-1^pPROX^ interface. The protein is colored using the same color scheme as in Fig. 3, and residues participating in aromatic interactions are shown as sticks. (**E**) The generalized correlation–based dynamical network analysis (*79*) was used to extract correlations of motions between SdrD and DSG-1^pPROX^interface. The heatmap matrix shows highly correlated interface residues (correlation values > 0.2). (**F**) SdrD:DSG-1^pPROX^. Highly correlated residues colored according to the heatmap in (E). We observe that most residues are located on SdrD latch motif and also at EC4 interface with the latch. Residues participating on aromatic interactions are labeled.

Next, we investigated the loading rate dependence of SdrD adhesion to both corneocytes and the DSG-1 protein using dynamic force spectroscopy (DFS) with AFM pulling experiments at varying speeds, providing different loading rates (Fig. 5, A and B). Consistent with the BE (*72*–*74*) and Dudko-Hummer-Szabo (DHS) models (*75*), our initial results demonstrated that higher loading rates led to increased rupture forces. Notably, experiments with corneocytes at high loading rates revealed a population resisting adhesion forces up to 4 nN (Fig. 5A), indicating that mechanical stress enhances bacterial-corneocyte adhesion. Similarly, we observed that the strength of the SdrD–DSG-1 bond also increases with applied mechanical force (Fig. 5B).

The DFS plot of the corneocyte experiments (Fig. 5A) shows that the 2-nN forces fit a BE model with a kinetic off rate (*k*_off_) of 8.8 × 10^−8^ s^−1^ and a transition energetic barrier at (*x*_β_) of 3.40 × 10^−2^ nm. However, at higher loading rates, we observed a transition to a high-force regime in the 3- to 5-nN range, which exceeds BE model predictions. For the SdrD–DSG-1 interaction (Fig. 5B), the DFS adhesion and unfolding plots fitted the BE model, yielding kinetic parameters with a *k*_off_ of 1.4 × 10^−7^ s^−1^ and a transition energetic barrier at (*x*_β_) of 3.96 × 10^−2^ nm, matching those on corneocytes. At higher loading rates, the high-force adhesion population was even more pronounced with DSG-1.

This shift suggests a mechanical activation of the adhesion complex, allowing it to withstand higher forces under stress. Such force-enhanced adhesion may provide a critical advantage for *S. aureus* during skin colonization, helping it resist mechanical challenges like hydrodynamic flow, cell-surface contacts, and epithelial turnover, confirming that mechanical stress activates a strong SdrD–DSG-1 interaction. While the 3- to 5-nN adhesion forces observed in corneocytes could involve multiple adhesins, this is unlikely for the SdrD–DSG-1 experiments, where DSG-1 was identified as the specific ligand for SdrD. In these conditions, catch-bonds formed by SdrD with DSG-1 strengthen under tension, marking this interaction as the strongest noncovalent bond recorded to date at 10^5^ pN/s. This behavior is consistent with previous findings for SdrG using magnetic tweezers (*76*), but the forces observed here, particularly the 4 nN regime, set a benchmark for noncovalent interactions.

### Atomic-level insights into the hyperstability of the SdrD complex under mechanical load

To elucidate how the SdrD:DSG-1 interaction could become more resilient to shear forces than the SdrG:Fgβ complex, we used our in silico SMFS approach, which allows us to track every atom of the system during the rupture event. To ensure the computational mechanism matched the experimental observations, we built a DFS using both aa-SMD and CG-SMD simulations (Fig. 5C). A total of 560 replicas were simulated for a combined duration of ~871 μs (tables S2 and S3). Simulations were performed on DGX-A100 nodes using NAMD (aa-SMD) and GROMACS (CG-SMD), following established protocols (*77*). The in silico DFS showed the same trend as the experimental work, with increasing mechanical force resilience for high force regimes (Fig. 5C).

To gain atomic-level insights into the DFS results, we analyzed the aa-SMD trajectories using standard protocols in Visual Molecular Dynamics (VMD) software (*78*) and the dynamical network analysis approach (*79*). In MD simulations, dynamical network analysis helps identify communication pathways and correlated motions between atoms or residues in biomolecules. Nodes, typically amino acid residues, are connected by edges representing interactions, which are filtered by distance and weighted based on correlation strength, using mutual information theory to capture communication between nodes (fig. S9). Within this network, force propagation pathways can be visualized (*80*) between the SMD anchor and the pulling points (fig. S10). Our analysis revealed that most of the force propagates from the protein latch through the peptide, indirectly interacting with β sheets in the N3 domain. This force transmission pattern differs from those observed in SdrG (*35*, *36*) and Bbp (*34*), where connections were observed between the latch and the N2 domain.

Community analysis of the network (fig. S11) highlights key amino acid residues with strong interconnections. In dynamical network analysis, communities are groups of tightly connected nodes (residues) that interact more with each other than with the rest of the network. Identifying these communities reveals functional regions within biomolecules, such as domains or allosteric sites. In our case, the latch and N2 domain were grouped in the same community (purple), although the connection was less pronounced than what was previously observed for SdrG and Bbp. We identified a unique community that links the latch and EC4, as well as a central community connecting the peptide, latch, and N3 domain (salmon). This previously unidentified pattern of highly connected residues between the peptide, latch, and cadherin EC4 domain supports the hypothesis that EC4 enhances the rigidity and stability of the complex under high-force loads. These findings are further supported by the network betweenness analysis (fig. S12). Betweenness is a measure of the importance of a node (residue) in facilitating communication within the network. High betweenness values indicate that a residue plays a critical role in connecting different regions of the protein. In our analysis, the strong correlation between the N2 and N3 domains with EC4, as indicated by the betweenness values, underscores the pivotal role of EC4 in maintaining the structural integrity of the complex under mechanical stress.

To investigate further which residues contribute the most for the hyperstability of the complex, we can plot the pairwise residue interactions at the complex interface (Fig. 5, E and F). Previous studies have shown that SdrG:Fgβ exhibits catch-bond behavior (*35*, *76*), which is also expected for SdrD:DSG-1. Highly connected interfaces have been associated with binding energy and also with mechanical resilience properties (*39*, *40*). Our results show that the highest correlated residues are located at the interface between SdrD and the EC4 domain, followed by interactions between the SdrD latch and p_PROX_ (Fig. 5E and fig. S13).

Visual inspection of the SMD trajectories revealed that SdrD residues Tyr^361^, Pro^533^, and Phe^557^, along with DSG-1 residue Phe^486^, are involved in Π-stacking interactions that persist until just before the complex ruptures (Fig. 5D). Additionally, Pro^533^ and Tyr^361^ are structurally aligned with key residues from the ClfB binding site, which are important for ligand binding (*33*), and both are highly connected at the ligand interface. In all, the simulations revealed that, unlike the previously studied SdrG:Fgβ interaction, which is primarily dominated by backbone-backbone hydrogen bonds, the SdrD:DSG-1 complex is further stabilized by Π-stacking of aromatic residues as well as interactions between EC4 and SdrD.

### Calcium’s role in enhancing the mechanostability and adhesion of SdrD complex

**Fig. 6.**
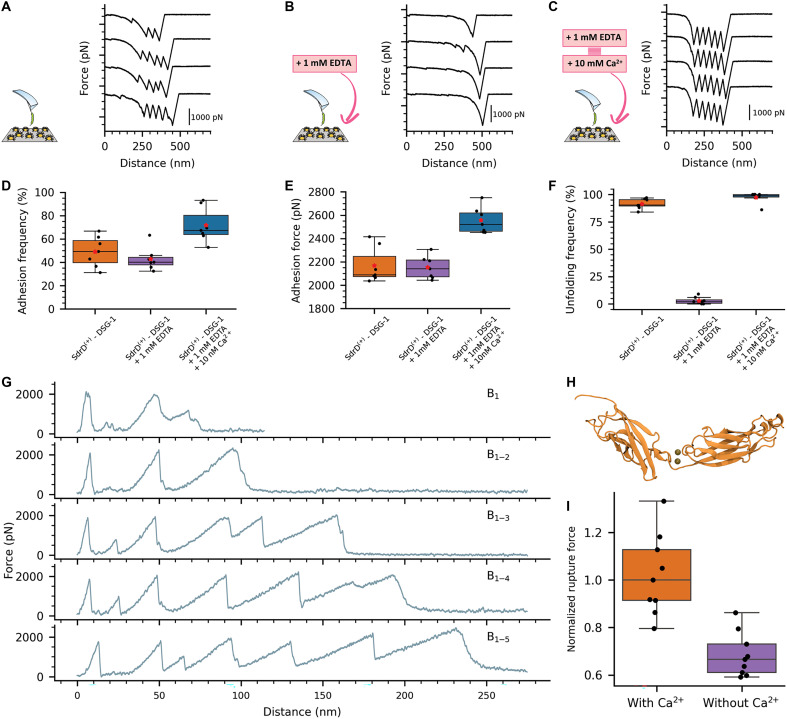
Calcium plays a crucial role in SdrD A-domain mechanostability. The mechanoproperties of SdrD are modulated by calcium ions. As before, SMFS experiments using AFM tips functionalized with DSG-1 interacting with *L. lactis* SdrD^(+)^ were performed in presence of calcium (**A**), when calcium was removed through chelation (**B**) and also in further addition of a higher calcium concentration (**C**). (**D**) Adhesion frequencies (D) and adhesion forces (**E**) observed for the experiment in a higher calcium concentration were unexpectedly higher. Unfolding forces associated with the B-domains were abolished when calcium was removed (**F**). Events with no adhesion were not considered on the boxplots; only adhesion forces of >1000 pN were considered. (**G**) Force-versus-extension curves for SMD simulations where SdrD B-domains were sequentially added to the protein structure. We notice no substantial changes on the rupture forces of the last peak, associated with breaking the interaction between SdrD and DSG-1^pPROX^, which leads us to believe that the calcium associated with DSG-1 EC domains, instead of the ones present on SdrD B-domains, might be involved on the experimentally observed increase on the adhesion forces. (**H**) Schematic representation of a toy system created to evaluate the role of DSG-1 EC domain calcium ions. EC3 and EC4 3D structure is represented in cartoon while calcium ions that stabilize the interface are represented as tan spheres. (**I**) Normalized unfolding forces measured over SMD simulations where we pull EC4 through a constricted pore region to mimic the bulk resistance offered by EC4 adjacent to p_PROX_ with our aa-SMD simulations. We observe a major difference on the unfolding forces in presence of calcium ions at the interface stabilizing the EC3:EC4 interaction, suggesting that both calcium and the location of the binding site might be playing a role in the complex hyperstabilization. Red stars on (D) to (F) represent average points.

Another important aspect of the adhesion mechanism is its known calcium dependence (*81*, *82*). Protein folds or complexes with nanonewton-range mechanostability typically include calcium in their structure (*82*–*84*). For adhesins, B-domains are known to be highly dependent on calcium, with their resilience to mechanical unfolding becoming negligible when calcium ions are depleted (*67*). Similarly, we observed that calcium plays a crucial role in the mechanostability of the SdrD B-domains. SdrD binds calcium ions through its B1-B5 domains (*31*, *32*). Ca^2+^ binding induces conformational changes in SdrD, proposed to regulate the distance between the bacterial surface and the A-domain in a spring-like fashion, ensuring optimal ligand-binding. Here, we assessed the extent to which calcium modulates the mechanical properties of SdrD using SMFS with DSG-1–modified tips. Unfolding forces (sawtooth patterns) associated with the B-domains in normal conditions (Fig. 6A) were abolished (unfolding frequency dropping from 91 to 5%) when calcium was removed via chelation (1 mM EDTA) (Fig. 6B). Adding 10 mM Ca^2+^ restored the sawtooth patterns (Fig. 6C), and the high concentration of Ca^2+^ created a more stable pattern, indicating the role of Ca^2+^-binding sites in each B-domain.

The adhesion frequency in these experiments was not negatively affected by Ca^2+^ depletion (Fig. 6D), and the adhesion force of 2156 ± 210 pN and rupture length associated with the A-domain were not altered (Fig. 6E and fig. S14). The addition of 10 mM Ca^2+^ showed an unfolding frequency of B-domains similar to the initial case but markedly different from the system where calcium was depleted (Fig. 6F). We speculate that, under standard conditions, SdrD binds calcium and possibly other ions present in the growth medium in low amounts, whereas adding 10 mM Ca^2+^ following chelation enables all B-domain binding sites to interact with this ion, contributing to their optimal folded conformation, which is illustrated by the full unfolding signature (five peaks + one adhesion peak). Our observation that Ca^2+^-binding sites control the mechanostability of SdrD B-domains is consistent with SMFS on purified B1 domains from the structurally related SdrG protein.

Additionally, we found that Ca^2+^ greatly enhances the binding properties of the A-domain, with the adhesion frequency and force increasing from 49 to 71% and 2076 ± 248 to 2601 ± 163 pN (658 adhesion force profiles; seven different cells), respectively (Fig. 6, D and E, and fig. S15). This unexpected finding suggests that Ca^2+^ not only governs the stability of the SdrD B-domains but also enhances the ligand-binding strength of the A-domain. Previous studies showed that calcium enhances the stability of B-domains but had little effect on increasing A-domain binding strength (*67*). To investigate the role of B-domains in stabilizing the A-domain, we performed CG-SMD simulations where we sequentially removed B-domains. Results showed no major differences in A-domain rupture forces regardless of the number of B-domains present (Fig. 6G). Visual inspection of the aa-SMD trajectories revealed that the EC4 cadherin creates a bulk resistance point when dissociating SdrD:p_PROX_ under force. Therefore, we hypothesize that EC DSG-1 cadherin domains, stabilized by calcium ions, likely contribute to the increased binding strength in the A-domain. Between two adjacent cadherin domains, calcium ions stabilize the complex in a relatively rigid structure (Fig. 6H) (*85*). To test this, we created a toy system with p_PROX_, EC4, and EC3 cadherin domains, pulling p_PROX_ through a fixed nanopore to simulate cadherin unfolding under force and constriction like when p_PROX_ is bound to SdrD A-domain (fig. S16). Our results show that calcium-depleted cadherins are about 35% easier to unfold than those with calcium-bound sites (Fig. 6I), explaining the increased adhesion force observed experimentally.

### Elevated calcium concentrations in AD enhance SdrD-mediated adhesion, increasing bacterial binding across corneocyte surfaces

The increased mechanostability provided by Ca^2+^ may have important clinical implications, particularly in AD, where elevated Ca^2+^ concentration gradients are observed in affected cells. Filaggrin deficiency, a common feature in AD, leads to epidermal barrier dysfunction, reduced natural moisturizing factor levels, and abnormal distribution of corneodesmosomes across the corneocyte surface (*86*). Disruption of the epidermal barrier alters the calcium gradient, likely intensifying Ca^2+^-dependent interactions. Calcium gradients are also known to play a crucial role in epidermal differentiation (*87*).

**Fig. 7.**
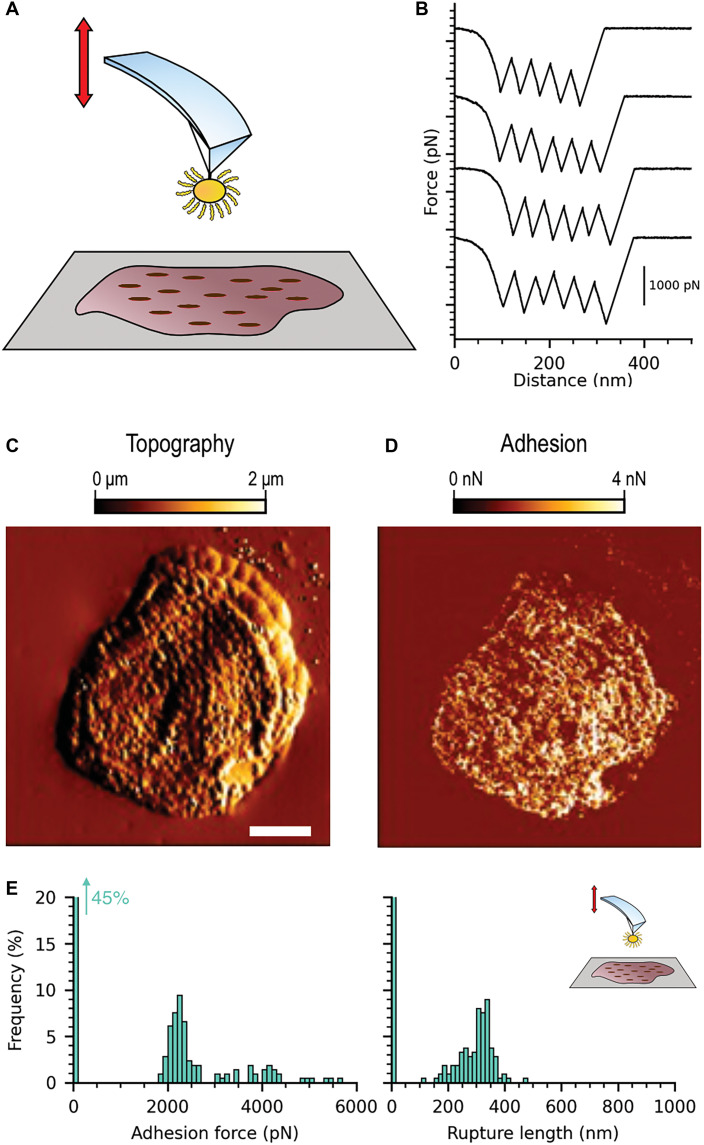
SdrD mediates bacterial adherence to AD corneocytes. (**A**) Schematic illustration of SCFS experiments, such as in Fig. 1A. This time, we mapped the molecular interactions between single *L. lactis* SdrD^(+)^ bacterial probe and corneocytes from the SC of subjects with AD. (**B**) Canonical sawtooth pattern detected on SCFS experiments for *L. lactis* SdrD^(+)^ binding to AD corneocytes. (**C** and **D**) Representative topographic images of the structure and adhesion of AD corneocytes, respectively. Different from healthy corneocytes, the strong rupture events (bright pixels) cover the entire cell surface. (**E**) Adhesion rupture forces were extremely strong, with a sharp distribution centered at 2156 pN and a second one around 4000 pN. Rupture lengths are around 315.8 nm. A representative histogram is shown (*n* = 212).

To explore the influence of Ca^2+^ and the molecular effects of SdrD adhesion in AD corneocytes, we conducted a new set of SCFS experiments (Fig. 7A). Previous studies have shown that AD corneocytes exhibit abnormal behavior of corneodesmosomal proteins, such as CDSN, which becomes diffused across the cell surface rather than remaining confined to the periphery (*88*). Immunofluorescence microscopy has also revealed an abnormal distribution of DSG-1, forming dense, diffuse patterns across the corneocyte surface (*18*). To test our hypothesis that DSG-1 follows a similar distribution pattern when interacting with *S. aureus* SdrD, we repeated the SCFS experiments using AD corneocytes (Fig. 7A). As in the previous experiments with *L. lactis* SdrD^(+)^ (Fig. 4A) binding to healthy corneocytes, the force measurements displayed the characteristic sawtooth pattern (Fig. 7B).

The first notable difference between healthy and AD samples was the texture of their surface. Topographic images of AD corneocytes (Fig. 7C) revealed small, circular protrusions, a few hundred nanometers in size, termed circular nano-objects (*89*), which are hypothesized to correspond to corneodesmosomes (*90*). The second clear distinction was in the distribution of adhesive events. While *S. aureus* primarily adhered to the edges of healthy corneocytes, it was able to target the entire surface of AD corneocytes (Fig. 7D). This distribution of adhesion events supports our hypothesis, as there is a clear correlation between the location of corneodesmosomes and the adhesion events. Another key observation was the increase in adhesion rupture forces (Fig. 7E and fig. S17). A distinct distribution was noted, with one peak centered at 2156 pN and a second peak around 4000 pN. Rupture lengths were ~321 ± 50 nm, consistent with the length of the adhesin (Fig. 7E and fig. S17).

**Fig. 8.**
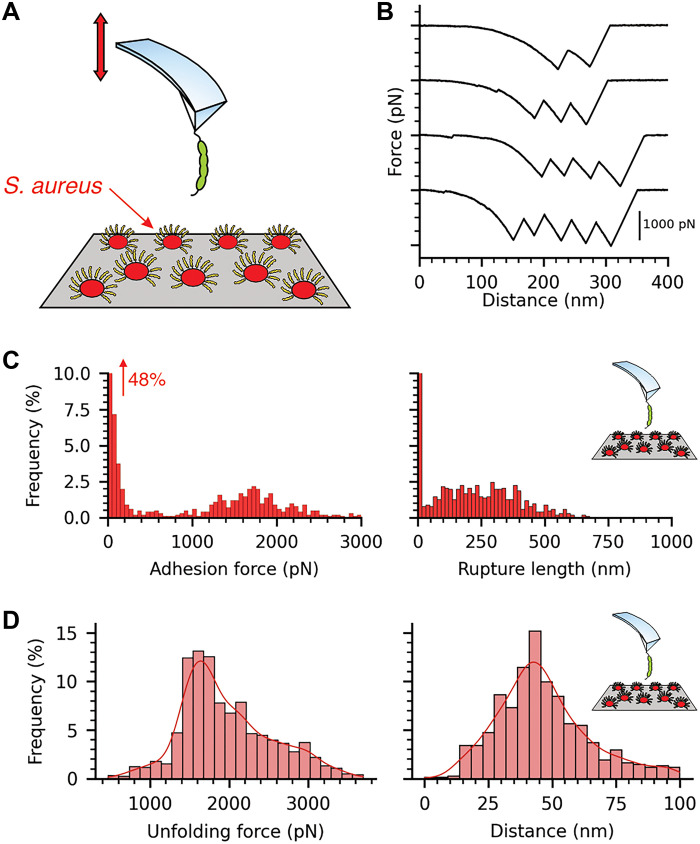
*S. aureus* AD08 strain isolated from a patient with AD shows ultrastrong forces. (**A**) Schematic illustration of SMFS experiments where we mapped the molecular interactions between *S. aureus* AD08 and AFM tips functionalized with DSG-1. (**B**) Similar to *L. lactis* SdrD^(+)^, sawtooth patterns reflecting unfolding of SdrD B-domains were observed. (**C**) Adhesion rupture forces were equally extremely strong, with a sharp distribution around 1546 pN, and rupture lengths are around 200 to 300 nm, consistent with the adhesin length. A representative histogram is shown (*n* = 1019). (**D**) Unfolding force and distance for the *S. aureus* AD08:DSG-1 SMFS experiments. (*n* = 1234).

Last, we performed SMFS experiments to investigate the interaction between DSG-1 and *S. aureus* clinical isolates. We used the AD08 strain, which was isolated from the infected skin lesion of a patient with AD (Fig. 8A) (*28*, *91*). The DSG-1:AD08 interaction exhibits two distinct rupture force populations, with a main peak centered at 1546 ± 307pN, similar to what was observed for *L. lactis* expressing SdrD, and rupture lengths between 284 ± 142 nm, consistent with the expected size of the adhesin (Fig. 8C and fig. S18). Additionally, we observed characteristic sawtooth patterns indicative of SdrD B-domain unfolding, with peak-to-peak distances of ~40 nm (Fig. 8B). The unfolding force and extension profiles closely match those seen in DSG-1:SdrD^(+)^ *L. lactis* interactions (Fig. 8D), strongly supporting that SdrD is the adhesin mediating binding to DSG-1 in the AD08 strain.

## DISCUSSION

Our study reveals the mechanism by which *S. aureus* uses the SdrD protein to bind corneocytes, identifying DSG-1 as a key molecular target (Fig. 2). Using a combined experimental and computational biophysics approach, ranging from physiologically relevant pathogen-skin interactions to atomistic molecular analyses, we identified two potential binding sites on DSG-1. We confirmed that SdrD specifically interacts with the region between the transmembrane and EC4 domains of DSG-1 (Fig. 3), as demonstrated by both in silico predictions and experimental SMFS assays. This interaction displays remarkable strength, which is further enhanced under mechanical load. The results are consistent with catch-bond behavior, where the bond lifetime increases in response to applied force.

Notably, the mechanostability of the SdrD complex under high Ca^2+^ concentration exceeds that of previously known SdrG interactions, making it the strongest protein-protein interaction reported to date (Fig. 5). The pivotal role of the EC4 cadherin domain in this enhanced stability provides previously unidentified clues into the structural dynamics of bacterial-human adhesion. Unlike SdrG, which primarily depends on backbone-backbone hydrogen bonds (*35*), SdrD uses additional interactions with DSG-1’s EC4 domain and a π-stacking of amino acids near its latch (Fig. 5). Key residues involved in these interactions, such as Tyr^361^, Pro^533^, and Phe^557^ in SdrD and Phe^486^ in DSG-1, are crucial for maintaining the integrity of the complex. This structural arrangement contributes to the exceptional strength of the SdrD:DSG-1 binding.

The critical contribution of the E-cadherin domains to complex stability was confirmed by both in vitro and in silico SMFS experiments. In both approaches, a truncated DSG-1 peptide lacking the EC4 domain was still able to bind to SdrD; however, the rupture forces were significantly reduced compared to the full-length protein (fig. S19). Notably, even in the absence of the E-cadherin domains, the complex resisted forces in the nanonewton regime. These results underscore a key distinction from SdrG, which binds exclusively to a similar peptide region, demonstrating that SdrD enhances its binding strength through a dual mechanism: forming additional contacts with the EC4 domain and establishing a stabilizing π-stacking interaction near its latch. This extended interaction network appears to be essential for the exceptional mechanostability of the SdrD:DSG-1 complex.

Furthermore, calcium ions are essential for the proper folding and stability of many MSCRAMMs, including SdrD (*26*, *32*, *33*, *67*, *92*, *93*). In this work, we showed that calcium ions play a fundamental role in SdrD complex stability by stabilizing the E-cadherin domains. Specifically, Ca^2+^ reinforces the EC3:EC4 interface, which, in turn, strengthens the EC4 fold and enhances the overall stability of the SdrD:DSG-1 complex (Fig. 6). Our experiments demonstrated that calcium depletion significantly weakened the mechanostability of the complex, while reintroducing Ca^2+^ not only restored but also further enhanced the bond’s strength. Given that calcium gradients play a crucial role in epidermal differentiation, Ca^2+^ may contribute to increased virulence of *S. aureus* infections under certain cell conditions. In damaged skin, the disrupted barrier alters the calcium gradient, potentially amplifying these interactions and bacterial adhesion capabilities (*87*).

Beyond its role in host protein integrity, calcium is also known to be important for bacterial virulence more broadly (*94*). Disrupting calcium binding in these proteins could weaken the structure and function of multiple adhesins simultaneously, thereby reducing *S. aureus* adhesion and colonization. In addition to that, monoclonal antibodies targeting DSG-1’s EC domains, especially EC4, could serve as competitive inhibitors that block SdrD binding without compromising DSG-1’s role in host adhesion. Similar therapeutic approaches have been explored against other bacterial adhesins, such as anti-ClfA antibodies designed to prevent *S. aureus* binding to fibrinogen. While these strategies have shown limited success in clinical settings (*95*–*97*), targeting DSG-1 might offer a more specific and accessible intervention point, especially in skin conditions like AD where SdrD adhesion is more prominent. This highlights the potential for targeting calcium-dependent interactions as a broader antimicrobial strategy.

At the tissue level, our findings demonstrated that *S. aureus* exploits the altered skin environment in AD to enhance adhesion and possibly increase colonization by *S. aureus*. SCFS experiments with AD corneocytes revealed increased adhesion frequencies at higher rupture forces, likely due to the abnormal, diffuse distribution of DSG-1 on the cell surface (Fig. 7). The same experiments were performed with *S. aureus* AD08 strain, confirming the role of SdrD:DSG-1 interaction on a clinically relevant bacterial strain (Fig. 8). While *S. aureus* is not the primary cause of this disorder, it is the most common pathogen associated with AD-related infections, exacerbating the condition. This worsening effect is likely driven by increased SdrD binding to the corneocyte surface, facilitated by the disrupted corneodesmosomal architecture.

In summary, our study provides a comprehensive understanding of the molecular mechanisms underlying *S. aureus* adhesion to human skin, identifying DSG-1 as a critical ligand for SdrD. We demonstrated the remarkable catch-bond behavior of this interaction under mechanical stress and the essential role of calcium in stabilizing the complex. Additionally, the enhanced adhesion to AD corneocytes reveals how *S. aureus* exploits compromised skin conditions to strengthen its colonization at the molecular level. These findings not only expand our knowledge of bacterial adhesion but also open possible avenues for targeted therapies against *S. aureus* infections and skin disorders like eczema. Our study highlights the discovery of the strongest known protein-protein interaction, driven by a previously unknown, calcium-enhanced DLL mechanism, representing a major advancement in the field of bacterial pathogenesis and mechanobiology.

## METHODS

### Experimental setup

#### Bacterial strains and growth conditions

Stationary phase cultures of *L. lactis* strain MG1363 carrying the pKS80 plasmid containing the SdrD gene, pKS80::sdrD [SdrD^(+)^] (*98*), or *L. lactis* strain transformed with the empty pKS80 plasmid [SdrD^(−)^] (*98*) were obtained from Foster and coworkers (*98*). Cultures were grown overnight in brain heart infusion (BHI) media in the presence of erythromycin (10 μg/ml) with shaking (180 rpm) at 30°C. Bacteria were then collected by centrifugation (5 min at 2000*g*), washed twice with phosphate-buffered saline (PBS), and resuspended in PBS, with a 50-fold dilution. A small amount (50 μl) of the suspension was then deposited on the left side of a polystyrene petri dish, and the cells were allowed to adhere for 10 min then washed with PBS. Confirmation that functional SdrD was present on the surface of *L. lactis* pKS80::sdrD was achieved using in vitro adherence essays with DSG-1, (see fig. S2). *L. lactis* pKS80 served as negative control. The experiment was performed in three biological replicates; a multicomparison using two-way analysis of variance (ANOVA) statistical test was performed with a Šidák posttest (*P < 0.05).

#### Corneocytes collection

Healthy and AD corneocytes were collected using a tape stripping method. Tape strips were collected from healthy participants aged 18 to 65 or pediatric patients with AD aged under 18 years. Sex, gender, age, or ethnicity was not recorded. The study was conducted in accordance with the Declaration of Helsinki and was approved by the Research Ethics Committee of Children’s Health Ireland at Crumlin, Dublin, Ireland (ref. 2020402). The collection of tape strips from children with AD was approved by the Research Ethics Committee of Our Lady’s Children’s Hospital, Crumlin, Dublin, Ireland.. Written informed consent from parents/guardians and patient assent (where feasible) was obtained before study enrolment. A clinically unaffected site on the volar forearm was used for SC sampling. Circular adhesive tape strips (3.8 cm^2^; D-Squame; Monaderm, Monaco, France) were attached to the skin and pressed for 10 s using a D-Squame pressure device (D500; CuDerm, Dallas, TX) with a constant pressure (225 g/cm^2^) and then removed using tweezers. Eight consecutive tape strips were removed from the same site and immediately stored at −70°C until analysis. The sixth and seventh tape strips were used for this study. On the right side of the dish, a piece of tape strip (5 mm by 5 mm) was immobilized using double-sided tape. The petri dish was filled with 3 ml of PBS.

#### AD08 collection

*S. aureus* AD08 is a clonal complex isolate 1 isolated from an infected skin lesion of a patient with AD (*99*). Overnight cultures of *S. aureus* were grown in 10 ml of tryptic soy broth (TSB) with shaking (180 rpm) at 37°C. Bacterial cells were harvested via centrifugation (2000*g* for 5 min) and washed once with TSB medium. They were adjusted to an optical density at 600 nm (OD_600_) of 0.05 and cultured until reaching the late exponential phase (OD_600_ = 0.8) in TSB. Last, the cells were harvested by centrifugation and prepared as described for *L. lactis*.

#### Single-cell force spectroscopy

Colloidal probes were prepared as described below. Under the control of a NanoWizard III atomic force microscope (JPK Instruments) coupled to an optical microscope, a triangular-shaped tipless cantilever was brought manually into contact with a small droplet of ultraviolet (UV)–curable glue, spread on one side of a glass slide, and then approached to catch a single silica microsphere (6-μm diameter, Bangs Laboratories Inc.) deposited on the other side of the glass side. The colloidal probe was then exposed to a UV lamp for 15 min to cure the glue. Last, to be able to electrostatically immobilize a single bacterial cell, the colloidal probe was incubated in a 10 mM tris-HCl buffer solution (pH 8.5) containing dopamine hydrochloride (4 mg ml^−1^) for an hour and washed in the same buffer. The nominal spring constant of these cantilevers was determined by the thermal noise method, giving an average value of ~0.08 N m^−1^. To perform the AFM experiment, the colloidal probe was first brought into contact with a single isolated bacterium (localized by optical microscopy) to catch it electrostatically through dopamine. The obtained so-called cell probe was then positioned over the corneocyte surface. Force-distance curves were collected in QI mode using a constant speed of 30 μm s^−1^, a ramp length of 1 μm, an applied force of 500 pN, a dwell time of 0 s and a *Z* closed loop. Maps of 256 × 256 pixels were recorded on 50 μm–by–50 μm areas of the corneocyte surface at room temperature. Then, force-distance curves were collected in FV mode using a constant approach and retraction speed of 1 μm s^−1^ and 0.5 to 10 μm s^−1^ (DFS), respectively, a ramp length of 1 μm, an applied force of 250 pN, a dwell time of 0 s, and a *Z* closed loop. Maps of 16 × 16 pixels were recorded on 5 μm–by–5 μm areas of the corneocyte surface (usually, on two different spots of the surface, to account for variability in ligand coverage) at room temperature. Number of cells and details on the analyzed data are included at table S1.

#### Functionalization of tips with DSG-1

Gold cantilevers (PNP-TR probes, Pyrex Nitride Probe with TRiangular Cantilevers, from NanoWorld) were immersed overnight in an ethanol solution containing 1 mM of 10% 16-mercaptododecahexanoic acid/90% 1-mercapto-1-undecanol (Sigma-Aldrich), rinsed with ethanol, and dried with N_2_. Cantilevers were then immersed for 30 min into a solution containing *N*-hydroxysuccinimide (NHS; 10 mg ml^−1^) and 1- ethyl-3-(3-dimethylaminopropyl)-carbodiimide (25 mg ml^−1^) (Sigma-Aldrich) and rinsed with ultrapure water. Last, they were incubated with recombinant human DSG-1 (0.1 mg ml^−1^; Gentaur) for 1 hour, rinsed further with PBS buffer, and then immediately used without dewetting.

#### *Functionalization of tips with* $p_{\text{PROX}}^{*}$

The MSCT-D cantilevers (Bruker) were first amino-functionalized using the gas-phase method of reaction with 3-aminopropyltriethoxysilane as reported previously (*100*). The functionalization was done as previously described (*48*, *101*, *102*). Briefly, the amino-functionalized cantilevers were initially immersed for 2 hours in a reaction solution prepared by dissolving 1 mg of NHS-PEG27-maleimide (JKU, Linz, Austria) in 0.5 ml of chloroform and mixed with 30 μl of triethylamine. Following immersion, the cantilevers were rinsed with chloroform and dried under an argon stream. The cysteine-bearing peptide ININIQSFGNDDRTNTEPNGSGSGSGSC, here called $p_{\text{PROX}}^{*}$ , is based on p_PROX_ sequence, considering the amino acids in direct contact with SdrD’s latch domain. The peptide was procured from GenScript. A 100-μl solution of $p_{\text{PROX}}^{*}$ (100 μg ml^−1^) was sequentially mixed with 2 μl of EDTA (100 mM, pH 7.5), 5 μl of Hepes (1 M, pH 7.5), 2 μl of tris(2-carboxyethyl)phosphine (TCEP) hydrochloride (100 mM), and, lastly, 2 μl of Hepes (1 M, pH 9.6). This prepared mixture was then dispensed onto the cantilevers. After a subsequent 2-hour incubation, the cantilevers were thoroughly washed three times with PBS.

#### Single-molecule force spectroscopy

A 100× diluted overnight bacterial suspension (50 μl) was deposited on a polystyrene petri dish, and the cells were allowed to adhere for ~15 min before being washed with PBS and the petri dish being filled with PBS, similar to SCFS experiments. DSG-1–functionalized gold cantilevers, initially calibrated by the Thermal tune method (*k* ~ 0.06 N m^−1^), were then brought into contact with the surface of isolated single bacterial cell to record force-distance curves in the AFM force volume mode. Measurements were performed at room temperature with a NanoWizard III or IV AFM. We recorded 32 pixel by 32 pixel maps on areas of 0.5 μm–by–0.5 μm on the bacterial surface, with a constant approach and retraction speed of 1 μm s^−1^, a ramp length of 1 μm, an applied force of 250 pN, a dwell time of 0 s, and a *Z* closed loop, exactly as in SCFS measurements. For loading rate experiments, the retraction speed was varied: 0.5, 1, 3, 5, and 10 μm s^−1^. The number of cells and details on the analyzed data are included at table S1.

#### Calcium experiment

After the recording of maps in the normal conditions, SMFS experiments were repeated in presence of 1 mM EDTA (Sigma-Aldrich) in the medium after 15 min of incubation. Then, a CaCl_2_ solution (Sigma-Aldrich) was injected at a final concentration of 10 mM in the medium. After 15 min of incubation, similar force-distance curves were recorded. Number of cells and details on the analyzed data are included at table S1.

#### Data analysis

Initial data analysis was carried out with data processing software from JPK Instruments (Berlin, Germany). Unfolding and adhesion peaks were well fitted using the worm-like chain model. All plots and final analysis were performed with in house python scripts. Adhesion events were considered for all measurements with force values of >0 pN, unless mentioned. Kernel density estimators were applied to fitting of unfolding forces and distances (Figs. 4, B and C, and 8D. The BE model (*72*, *74*) was used to fit SCFS and SMFS experiments (Fig. 5, A and B); all the fitted parameters are available in the text or figure legends.

### Molecular modeling of the SdrD:DSG-1 interaction

#### Protein sequences

The protein sequences for the SdrD and DSG-1 protein EC1 to EC4 were obtained from UniProt (*103*) IDs Q2G0L4 (isoform 1) and Q02413, respectively.

#### Protein structure prediction

The prediction of the apo tridimensional structure of the DSG-1 EC domains (residues 1 to 500) was performed using AlphaFold2 (*104*). Twenty-four models were generated and optimized using AMBER (*105*) under the AlphaFold2 protocol. Other parameters were assigned as default. The models were ranked by the per-residue confidence metric called the predicted local distance difference test score (*106*), and only the top ranked model was used for further analysis. The full apo tridimensional structure of SdrD (residues 1 to 1349) was also predicted by AlphaFold2 using the same protocol described above

#### Prediction of the SdrD binding site on DSG-1 structure

The 3D structure model of DSG-1 revealed two loop regions, e.g., with no defined secondary structure: (i) region 1: at the N-terminal loop (residues 1 to 42); and (ii) region 2: between the membrane and the EC4 (residues 485 to 548). The secondary structure propensity to form β strand was calculated for both regions using JPred4 server (*107*). From region 1, we selected the residues 25 to 42, here called distal peptide or p_DIST_. The secondary structure prediction for region 2 did not reveal any propensity to form strands or any defined secondary structure. Because this could happen once the peptide is already bound to the adhesin, we used an alternative strategy to select a peptide from the proximal region, here called p_PROX_. The crystal structure of SdrD in its apo form (*33*) revealed a similar ligand binding region with the available structures for ClfB. Region 2 protein sequence was then aligned with ClfB available ligands on the PDB (*108*): dermokine (PDB ID: 4f20), keratin (PDB ID: 4f1z), and Fgα (PDB ID: 4f27). Additionally, we included Fgß complexed with SdrG (PDB ID: 1r17). The sequence alignment was done using MUSCLE (*109*) with default parameters. Although there is no remarkable sequence similarity between the peptides sequences and DSG-1, they were clustered on the region between residues 485 and 494. One of the Phe residues from Fgβ was aligned with Phe^486^ from DSG-1. We the selected p_PROX_ from residues 485 to 500.

#### SdrD:DSG-1 structure modeling

Attempts to use AlphaFold2 to model the complex between SdrD A-domain (residues 244 to 570) and DSG-1 with either p_DIST_ or p_PROX_ did not yield accurate conformations for the interface region between the latch and the peptide, so we decided for a comparative modeling approach using MODELLER (*57*). Because the available crystal structures for SdrD are in the apo form (*33*), an additional template was needed. Protein BLAST (*110*) was used to search for SdrD homologs against the PDB. SdrG (PDB ID: 1r17) complexed with Fgß was selected as the additional template with good sequence coverage to SdrD (98%) and *E*-value = 1^−24^). MODELLER v10.4 was used to build the complex using SdrG and SdrD templates (PDB ID: 4JE0). The calcium ion available at the SdrG crystal structure was transferred to SdrD model. Because Fgβ is not an ideal template for DSG-1 peptide, we used MODELLER special restraints function to enforce modeling the DSG-1 peptides as a β strand. Other parameters were assigned as default. A total of 20 models were generated for each system and ranked using the Discrete Optimized Protein Energy (DOPE) score (*111*). The SdrD:p_PROX_ model also included the EC4 domain, taking in consideration residues 378 to 500.

#### SdrD:DSG-1 full structure modeling

The 3D structure models for the full sequences of the SdrD:DSG-1 complexes where either p_DIST_ or p_PROX_ at the interface with SdrD A-domain was constructed using a combination of the previously described structures were generated using AlphaFold2 and MODELLER. In each case, the models were superimposed using the measure fit module available with VMD (*78*). For simplicity, we excluded some disordered regions from SdrD (residues 1 to 243 and 1130 to 1349). Two models were then created: (i) SdrD^244-1129^:DSG-1^123-500^: full structure where p_DIST_ is at the complex interface; and (ii) SdrD^244-1129^:DSG-1^378-500^: full structure where p_PROX_ is at the complex interface. To reduce the computational complexity of the system, we included only EC4 domain from DSG-1. Calcium atoms were modeled into the DSG-1 structure using MODELLER, according to the available structure of DSG-3 EC domains (PDB ID: 5eqx) (*112*). Because the calcium binding residues are highly conserved on adhesin B-domains (*33*), they were manually superimposed to all SdrD B-domains according to B1 calcium ions available at PDB ID 4JEZ.

#### aa-SMD simulations

Models of SdrDA-domain:DSG-1^pDIST^ and SdrDA-domain:DSG-1^pPROX^ were submitted to aa-SMD simulations using NAMD 3 (*58*) with the CHARMM36 (*113*) force field. In each case, the complex was solvated using the TIP3 water model (*114*), and the net charge of the protein was neutralized using a 150 mM salt concentration of sodium chloride. To preserve the models integrity, harmonic restraints were added to SdrD secondary structure through the extraBonds NAMD feature. All MD simulations were performed in periodic boundary conditions, and a distance cutoff of 11.0 Å was applied to short-range nonbonded interactions, whereas long-range electrostatic interactions were treated using the particle-mesh Ewald method (*115*). Before the aa-SMD, the system was submitted to an energy minimization protocol for 1000 steps. An MD simulation with position restraints in the protein backbone atoms was performed for 1 ns, with temperature ramping from 0 to 300 K in the first 0.5 ns at a time step of 2.0 fs in the NVT ensemble, which served to pre-equilibrate the system. aa-SMD simulations were performed in the NPT ensemble with temperature maintained at 300 K using Langevin dynamics for temperature and pressure coupling, the latter kept at 1 bar. The time step of integration was chosen to be 2 fs for all production runs. aa-SMD simulations were carried out in several replicas, using a constant velocity stretching protocol at different pulling velocities (table S2). SMD was used by harmonically restraining the position of the amino acid at the C terminus of SdrD and moving a second restraint point at the C terminus of DSG-1 peptide, with constant velocity in the *z* axis with a spring constant of 5 kcal/mol per square angstrom (3.475 N m^−1^). The force applied to the harmonic spring is then monitored during the time of the SMD. The pulling point was moved with constant velocity along the *z* axis, and, due to the single anchoring point and the single pulling point, the system is quickly aligned along the *z* axis. aa-SMD simulations were also performed for the SdrD:$p_{\text{PROX}}^{*}$ complex, using the same parameters as described above.

#### All-atom equilibrium MD simulations

Before binding energy calculations, the SdrDA-domain:DSG-1^pDIST^ and SdrDA-domain:DSG-1^pPROX^ models were submitted to equilibrium MD simulations using NAMD 3 with the CHARMM36 force field. System preparation, solvation, minimization, and equilibration runs followed the same protocol described above. Production runs were carried out for 100 ns in eight replicas for each system.

#### Free energy of binding prediction

The free energy of binding between SdrD and p_DIST_ or p_PROX_ was estimated using the MM-GBSA approach (*61*). MM-GBSA is based on MD trajectories of the complex. In summary, the free energy of the complex is estimated from the following equation

$$G=E_{\text{bnd}}+E_{\text{el}}+E_{\text{vdW}}+G_{\text{pol}}+G_{\text{np}}$$

where the first three terms are standard MM energy terms from bonded (bond, angle, and dihedral), electrostatic, and van der Waals interactions. $G_{\text{pol}}$ is obtained by using the GB model, whereas the nonpolar term is estimated from a linear relation to the solvent accessible surface area. In MM-GBSA, the entropy contribution to the binding energy is neglected. Implicit GBSA solvent models are used to estimate the solvation energies. MD snapshots were obtained from the last quarter of the MD trajectories. A bootstrap analysis was performed to assess the significance of the difference between the free energy of binding between SdrD and p_DIST_ or p_PROX_, accounting eight simulation replicas each. The data were resampled 1000 times with replacement to generate bootstrap samples. The bias-corrected and accelerated method was used to construct 95% confidence intervals for the mean difference between the two configurations. The difference between the two samples was estimated to be 3.80 U with 95% confidence interval (CI) of 3.51 for p_DIST_ and 95% CI of 3.51 for p_PROX_. The SE of the mean difference was calculated to be 0.15 U. The confidence intervals indicate that the mean difference between both peptides configurations is statistically significant.

#### CG-MD simulations

CG-MD simulations were performed for full models of the complex SdrD:DSG-1 with either p_DIST_, SdrD^244-1129^:DSG-1^23-500^, or p_PROX_, SdrD^244-1129^:DSG-1^378-500^, at the interface. We have also performed CG-MD simulations for the complexes containing only SdrD’s A-domain, SdrD^244-570^, and DSG-1^pDIST^ or DSG-1^pPROX + EC4(485-500)^, for a couple of very slow pulling velocities that would be too computationally expensive to simulate all-atom (table S3). In all cases, the protein complexes were converted onto the Martini 3.0 CG force field (v.3.0.b.3.2) (*116*) using Martinize2 v0.7.3 (*117*). A set of native contacts, based on the rCSU+OV contact map protocol, was computed from the equilibrated all-atom structure using the rCSU server (*118*) and used to determine Gō-MARTINI interactions (*119*) used to restraint the secondary and tertiary structures with the effective depth (epsilon) of Lennard-Jones potential set to 9.414 kJ mol^−1^. Gō-MARTINI interaction potential was multiplied by 10 for the protein residues at the interface with p_DIST_ or p_PROX_. All CG-MD simulations were performed using GROMACS (*70*) version 2021.5. The full model of SdrD:DSG-1 with p_DIST_ on the interface was placed into a rectangular box measuring 20 nm by 20 nm by 500 nm to the *x*, *y*, and *z* directions. The anchor point was set at the C terminus of EC4 (Gly^465^) and the pulling point was set at the C terminus of B5 domain (Asp^886^). Both points were used to align the protein to the *z* axis. Due to the size of the systems, smaller boxes and different pulling points were sampled to improve the computational time. The full model of SdrD:DSG-1 with p_PROX_ was placed into a rectangular box measuring 15 nm by 15 nm by 350 nm to the *x*, *y*, and *z* directions. The anchor point was set at the C terminus of B5 domain (Asp^886^), and the pulling point was set at p_PROX_ N-terminal residue (Ile^123^). Both points were used to align the protein to the *z* axis. The box was then solvated with Martini 3 water molecules. Systems were minimized for 10,000 steps with steepest descent, followed by a 10-ns equilibration at the NPT ensemble using the Berendsen thermostat at 298 K, while pressure was kept at 1 bar with compressibility set to 3 × 10^−4^ per bar, using the Berendsen barostat. A time step of 10 fs was used to integrate the equations of motion. Pulling simulations were subsequently done at the NVT ensemble with a time step of 20 fs. The temperature was controlled using the v-rescale thermostat (*120*) with a coupling time of 1 ps. For all CG-MD simulations, the cutoff distance for Coulombic and Lennard-Jones interactions was set to 1.1 nm (*121*), with the long-range Coulombic interactions treated by a reaction field (*122*) with ε*r* = 15. The Verlet neighbor search (*123*) was used in combination with the neighbor list, updated every 20 steps. The LINCS (*124*) algorithm was used to constrain the bonds and the leapfrog integration algorithm for the solution of the equations of motion. Several replicas of CG-SMD simulations were performed at a range of speeds described at table S3.

The CG-SMD simulations were systematically rescaled on the basis of the fraction of most probable loading rate and most probable force of the lowest overlapping pulling rate (5.0 × 10^−6^ nm/ps) between all-atom and CG simulations. Therefore, the CG loading rates and peak forces were multiplied by 6.11 and 1.89, respectively.

#### Analysis of SMD

For all simulations, the force-extension curves were analyzed by custom python scripts. For dynamic force spectra, the rupture or unfolding forces versus loading rate was plotted, and median forces and loading rates for each pulling speed were fitted to BE model (*72*, *74*) to estimate the effective distance to the transition state (Δ*x*) and the intrinsic dissociation rate or unfolding rate (*k*_off_) in the absence of force. The median forces and loading rates at the various pull rates were then used to fit a DHS model (*125*).

#### Dynamical network analysis

The generalized correlation–based dynamical network analysis (*79*) was used to extract correlations of motion between SdrD and DSG-1 interface residues over the aa-SMD simulations. We analyzed all replicas for the slowest pulling velocity (5.0 × 10^−6^ nm/ps). A network is defined as a set of nodes that represent amino acid residues, and the node’s position is mapped to that of the residue’s α carbon. Edges connect pairs of nodes if their corresponding residues are in contact, and two nonconsecutive residues are said to be in contact if they are within 4.5 Å of each other for at least 75% of analyzed frames. The mean correlation analysis was carried out on 500 frames before the chosen rupture events at each replica, using a cutoff of 0.2 for the mean correlation coefficients. Binding affinities were calculated using established protocols on the basis of the sum of correlations (*126*).

#### Images and plots

All protein structure renderings were performed using VMD (*78*, *127*), along with its plugins and TCL scripts, unless otherwise specified. Graphs were generated using Python 3 libraries, including Matplotlib (*128*), Pandas (*129*), and Seaborn (*130*).

## References

[1] A. M. Cole, S. Tahk, A. Oren, D. Yoshioka, Y. H. Kim, A. Park, T. Ganz, Determinants of *Staphylococcus aureus* nasal carriage. Clin. Diagn. Lab. Immunol. 8, 1064–1069 (2001). 11687441 10.1128/CDLI.8.6.1064-1069.2001 PMC96227

[2] A. F. Brown, J. M. Leech, T. R. Rogers, R. M. McLoughlin, *Staphylococcus aureus* colonization: Modulation of host immune response and impact on human vaccine design. Front. Immunol. 4, 507 (2014). 24409186 10.3389/fimmu.2013.00507 PMC3884195

[3] C. P. Parlet, M. M. Brown, A. R. Horswill, Commensal staphylococci influence *Staphylococcus aureus* skin colonization and disease. Trends Microbiol. 27, 497–507 (2019). 30846311 10.1016/j.tim.2019.01.008 PMC7176043

[4] A. M. Drucker, A. R. Wang, W. Q. Li, E. Sevetson, J. K. Block, A. A. Qureshi, The burden of atopic dermatitis: Summary of a report for the national eczema association. J. Invest. Dermatol. 137, 26–30 (2017). 27616422 10.1016/j.jid.2016.07.012

[5] J. M. B. Myers, G. K. K. Hershey, Eczema in early life: Genetics, the skin barrier, and lessons learned from birth cohort studies. J. Pediatr. 157, 704–714 (2010). 20739029 10.1016/j.jpeds.2010.07.009 PMC2957505

[6] J. A. Geoghegan, A. D. Irvine, T. J. Foster, *Staphylococcus aureus* and atopic dermatitis: A complex and evolving relationship. Trends Microbiol. 26, 484–497 (2018). 29233606 10.1016/j.tim.2017.11.008

[7] H. H. Kong, J. Oh, C. Deming, S. Conlan, E. A. Grice, M. A. Beatson, E. Nomicos, E. C. Polley, H. D. Komarow, NISC Comparative Sequence Program, P. R. Murray, M. L. Turner, J. A. Segre, Temporal shifts in the skin microbiome associated with disease flares and treatment in children with atopic dermatitis. Genome Res. 22, 850–859 (2012). 22310478 10.1101/gr.131029.111 PMC3337431

[8] J. J. Leyden, R. R. Marples, A. M. Kligman, Staphylococcus aureus in the lesions of atopic dermatitis. Br. J. Dermatol. 90, 525–530 (1974). 4601016 10.1111/j.1365-2133.1974.tb06447.x

[9] J. Sinagra, S. Pecetta, V. Bordignon, A. De Santis, L. Cilli, V. Cafiso, G. Prignano, B. Capitanio, C. Passariello, S. Stefani, P. Cordiali-Fei, F. Ensoli, Molecular and immunological characterization of *Staphylococcus aureus* in pediatric atopic dermatitis: Implications for prophylaxis and clinical management. J. Immunol. Res. 2011, 718708 (2011). 10.1155/2011/718708 PMC3205653 22110527

[10] M. Alsterholm, L. Strömbeck, A. Ljung, N. Karami, J. Widjestam, M. Gillstedt, C. Åhren, J. Faergemann, Variation in Staphylococcus aureus colonization in relation to disease severity in adults with atopic dermatitis during a five-month follow-up. Acta Derm. Venereol. 97, 802–807 (2017). 28374043 10.2340/00015555-2667

[11] A. L. Byrd, C. Deming, S. K. B. Cassidy, O. J. Harrison, W. I. Ng, S. Conlan, NISC Comparative Sequencing Program, Y. Belkaid, J. A. Segre, H. H. Kong, *Staphylococcus aureus* and *Staphylococcus epidermidis* strain diversity underlying pediatric atopic dermatitis. Sci. Transl. Med. 9, eaal4651 (2017). 28679656 10.1126/scitranslmed.aal4651 PMC5706545

[12] H. F. Chambers, F. R. DeLeo, Waves of resistance: *Staphylococcus aureus* in the antibiotic era. Nat. Rev. Microbiol. 7, 629–641 (2009). 19680247 10.1038/nrmicro2200 PMC2871281

[13] F. R. DeLeo, H. F. Chambers, Reemergence of antibiotic-resistant *Staphylococcus aureus* in the genomics era. J. Clin. Invest. 119, 2464–2474 (2009). 19729844 10.1172/JCI38226 PMC2735934

[14] C. P. Harkins et al., Methicillin-resistant *Staphylococcus aureus* emerged long before the introduction of methicillin into clinical practice. Genome Biol. 18, 130 (2017). 28724393 10.1186/s13059-017-1252-9 PMC5517843

[15] J. Y. Lee, I. R. Monk, A. Gonçalves da Silva, T. Seemann, K. Y. L. Chua, A. Kearns, R. Hill, N. Woodford, M. D. Bartels, B. Strommenger, F. Laurent, M. Dodémont, A. Deplano, R. Patel, A. R. Larsen, T. M. Korman, T. P. Stinear, B. P. Howden, Global spread of three multidrug-resistant lineages of staphylococcus epidermidis. Nat. Microbiol. 3, 1175–1185 (2018). 30177740 10.1038/s41564-018-0230-7 PMC6660648

[16] L. S. Miller, V. G. Fowler Jr., S. K. Shukla, W. E. Rose, R. A. Proctor, Development of a vaccine against *Staphylococcus aureus* invasive infections: Evidence based on human immunity, genetics and bacterial evasion mechanisms. FEMS Microbiol. Rev. 44, 123–153 (2020). 31841134 10.1093/femsre/fuz030 PMC7053580

[17] S. Eyerich, K. Eyerich, C. Traidl-Hoffmann, T. Biedermann, Cutaneous barriers and skin immunity: Differentiating a connected network. Trends Immunol. 39, 315–327 (2018). 29551468 10.1016/j.it.2018.02.004

[18] S. Igawa, M. Kishibe, M. Honma, M. Murakami, Y. Mizuno, Y. Suga, M. Seishima, Y. Ohguchi, M. Akiyama, K. Hirose, A. Ishida-Yamamoto, H. Iizuka, Aberrant distribution patterns of corneodesmosomal components of tape-stripped corneocytes in atopic dermatitis and related skin conditions (ichthyosis vulgaris, netherton syndrome and peeling skin syndrome type B). J. Dermatol. Sci. 72, 54–60 (2013). 23810772 10.1016/j.jdermsci.2013.05.004

[19] Y. Naoe, T. Hata, K. Tanigawa, H. Kimura, T. Masunaga, Bidimensional analysis of desmoglein 1 distribution on the outermost corneocytes provides the structural and functional information of the stratum corneum. J. Dermatol. Sci. 57, 192–198 (2010). 20116975 10.1016/j.jdermsci.2009.12.014

[20] C. M. Isacke, M. A. Horton, in *The Adhesion Molecule FactsBook*, C. M. Isacke, M. A. Horton, Eds. (Academic Press, ed. 2, 2000), pp. 80–82.

[21] C. Gismene, J. E. Hernández González, A. R. N. Santisteban, A. F. Ziem Nascimento, L. dos Santos Cunha, F. R. de Moraes, C. L. P. de Oliveira, C. C. Oliveira, P. Jocelan Scarin Provazzi, P. G. Pascutti, R. K. Arni, R. Barros Mariutti, *Staphylococcus aureus* exfoliative toxin e, oligomeric state and flip of p186: Implications for its action mechanism. Int. J. Mol. Sci. 23, 9857 (2022). 36077258 10.3390/ijms23179857 PMC9456352

[22] M. Amagai, J. R. Stanley, Desmoglein as a target in skin disease and beyond. J. Invest. Dermatol. 132, 776–784 (2012). 22189787 10.1038/jid.2011.390 PMC3279627

[23] H. F. Wertheim, M. C. Vos, A. Ott, A. van Belkum, A. Voss, J. A. J. W. Kluytmans, P. H. J. van Keulen, C. M. J. E. Vandenbroucke-Grauls, M. H. M. Meester, H. A. Verbrugh, Risk and outcome of nosocomial *Staphylococcus aureus* bacteraemia in nasal carriers versus non-carriers. Lancet 364, 703–705 (2004). 15325835 10.1016/S0140-6736(04)16897-9

[24] C. Von Eiff, K. Becker, K. Machka, H. Stammer, G. Peters, Nasal carriage as a source of *Staphylococcus aureus* bacteremia. N. Engl. J. Med. 344, 11–16 (2001). 11136954 10.1056/NEJM200101043440102

[25] T. J. Foster, J. A. Geoghegan, V. K. Ganesh, M. Höök, Adhesion, invasion and evasion: The many functions of the surface proteins of *Staphylococcus aureus*. Nat. Rev. Microbiol. 12, 49–62 (2014). 24336184 10.1038/nrmicro3161 PMC5708296

[26] T. J. Foster, The MSCRAMM family of cell-wall-anchored surface proteins of gram-positive cocci. Trends Microbiol. 27, 927–941 (2019). 31375310 10.1016/j.tim.2019.06.007

[27] C. Feuillie, C. Formosa-Dague, L. M. C. Hays, O. Vervaeck, S. Derclaye, M. P. Brennan, T. J. Foster, J. A. Geoghegan, Y. F. Dufrêne, Molecular interactions and inhibition of the staphylococcal biofilm-forming protein sdrc. Proc. Natl. Acad. Sci. U.S.A. 114, 3738–3743 (2017). 28320940 10.1073/pnas.1616805114 PMC5389287

[28] A. M. Towell, C. Feuillie, P. Vitry, T. M. da Costa, M. Mathelié-Guinlet, S. Kezic, O. M. Fleury, M. A. McAleer, Y. F. Dufrêne, A. D. Irvine, J. A. Geoghegan, *Staphylococcus aureus* binds to the N-terminal region of corneodesmosin to adhere to the stratum corneum in atopic dermatitis. Proc. Natl. Acad. Sci. U.S.A. 118, e2014444118 (2021). 33361150 10.1073/pnas.2014444118 PMC7817190

[29] F. Askarian, C. Ajayi, A. M. Hanssen, N. M. van Sorge, I. Pettersen, D. B. Diep, J. U. E. Sollid, M. Johannessen, The interaction between *Staphylococcus aureus* SdrD and desmoglein 1 is important for adhesion to host cells. Sci. Rep. 6, 22134 (2016). 26924733 10.1038/srep22134 PMC4770587

[30] R. M. Corrigan, H. Miajlovic, T. J. Foster, Surface proteins that promote adherence of *Staphylococcus aureus* to human desquamated nasal epithelial cells. BMC Microbiol. 9, 22 (2009). 19183486 10.1186/1471-2180-9-22 PMC2642834

[31] E. Josefsson, D. O’Connell, T. J. Foster, I. Durussel, J. A. Cox, The binding of calcium to the B-repeat segment of SdrD, a cell surface protein of *Staphylococcus aureus*. J. Biol. Chem. 273, 31145–31152 (1998). 9813018 10.1074/jbc.273.47.31145

[32] A. Y. Roman, F. Devred, V. M. Lobatchov, A. A. Makarov, V. Peyrot, A. A. Kubatiev, P. O. Tsvetkov, Sequential binding of calcium ions to the B-repeat domain of SdrD from *Staphylococcus aureus*. Can. J. Microbiol. 62, 123–129 (2016). 26639248 10.1139/cjm-2015-0580

[33] X. Wang, J. Ge, B. Liu, Y. Hu, M. Yang, Structures of SdrD from *Staphylococcus aureus* reveal the molecular mechanism of how the cell surface receptors recognize their ligands. Protein Cell 4, 277–285 (2013). 23549613 10.1007/s13238-013-3009-x PMC4875524

[34] P. S. Gomes, M. Forrester, M. Pace, D. E. Gomes, R. C. Bernardi, May the force be with you: The role of hyper-mechanostability of the bone sialoprotein binding protein during early stages of staphylococci infections. Front. Chem. 11, 1107427 (2023). 36846849 10.3389/fchem.2023.1107427 PMC9944720

[35] L. F. Milles, K. Schulten, H. E. Gaub, R. C. Bernardi, Molecular mechanism of extreme mechanostability in a pathogen adhesin. Science 359, 1527–1533 (2018). 29599244 10.1126/science.aar2094 PMC6451932

[36] M. C. Melo, D. E. Gomes, R. C. Bernardi, Molecular origins of force-dependent protein complex stabilization during bacterial infections. J. Am. Chem. Soc. 145, 70–77 (2023). 36455202 10.1021/jacs.2c07674

[37] R. dos Santos Natividade, M. Koehler, P. S. F. C. Gomes, J. D. Simpson, S. C. Smith, D. E. B. Gomes, J. de Lhoneux, J. Yang, A. Ray, T. S. Dermody, R. C. Bernardi, K. M. Ogden, D. Alsteens, Deciphering molecular mechanisms stabilizing the reovirus-binding complex. Proc. Natl. Acad. Sci. U.S.A. 120, e2220741120 (2023). 37186838 10.1073/pnas.2220741120 PMC10214207

[38] Q. Zhang, R. S. L. Rosa, A. Ray, K. Durlet, G. M. Dorrazehi, R. C. Bernardi, D. Alsteens, Probing SARS-CoV-2 membrane binding peptide via single-molecule AFM-based force spectroscopy. Nat. Commun. 16, 6 (2025). 39747000 10.1038/s41467-024-55358-9 PMC11696146

[39] M. S. Bauer, S. Gruber, A. Hausch, M. C. R. Melo, P. S. F. C. Gomes, T. Nicolaus, L. F. Milles, H. E. Gaub, R. C. Bernardi, J. Lipfert, Single-molecule force stability of the SARS-CoV-2–ACE2 interface in variants-of-concern. Nat. Nanotechnol. 19, 399–405 (2024). 38012274 10.1038/s41565-023-01536-7

[40] M. S. Bauer, S. Gruber, A. Hausch, P. S. F. C. Gomes, L. F. Milles, T. Nicolaus, L. C. Schendel, P. L. Navajas, E. Procko, D. Lietha, M. C. R. Melo, R. C. Bernardi, H. E. Gaub, J. Lipfert, A tethered ligand assay to probe SARS-CoV-2:ACE2 interactions. Proc. Natl. Acad. Sci. U.S.A. 119, e2114397119 (2022). 35312342 10.1073/pnas.2114397119 PMC9168514

[41] J. Seppälä, R. C. Bernardi, T. J. K. Haataja, M. Hellman, O. T. Pentikäinen, K. Schulten, P. Permi, J. Ylänne, U. Pentikäinen, Skeletal dysplasia mutations effect on human filamins’ structure and mechanosensing. Sci. Rep. 7, 4218 (2017). 28652603 10.1038/s41598-017-04441-x PMC5484675

[42] T. J. Haataja, R. C. Bernardi, S. Lecointe, R. Capoulade, J. Merot, U. Pentikäinen, Non-syndromic mitral valve dysplasia mutation changes the force resilience and interaction of human filamin a. Structure 27, 102–112.e4 (2019). 30344108 10.1016/j.str.2018.09.007 PMC6984350

[43] S. M. Sedlak, L. C. Schendel, H. E. Gaub, R. C. Bernardi, Streptavidin/biotin: Tethering geometry defines unbinding mechanics. Sci. Adv. 6, eaay5999 (2020). 32232150 10.1126/sciadv.aay5999 PMC7096159

[44] S. M. Sedlak, L. C. Schendel, M. C. R. Melo, D. A. Pippig, Z. Luthey-Schulten, H. E. Gaub, R. C. Bernardi, Direction matters: Monovalent streptavidin/biotin complex under load. Nano Lett. 19, 3415 (2018). 30346175 10.1021/acs.nanolett.8b04045 PMC6486461

[45] B. Yang, D. E. B. Gomes, Z. Liu, M. S. Santos, J. Li, R. C. Bernardi, M. A. Nash, Engineering the mechanical stability of a therapeutic complex between affibody and programmed death-ligand 1 by anchor point selection. ACS Nano 18, 31912–31922 (2024). 39514863 10.1021/acsnano.4c09220

[46] Y. F. Dufrêne, D. Martínez-Martín, I. Medalsy, D. Alsteens, D. J. Müller, Multiparametric imaging of biological systems by force-distance curve–based AFM. Nat. Methods 10, 847–854 (2013). 23985731 10.1038/nmeth.2602

[47] C. Formosa-Dague, Z. H. Fu, C. Feuillie, S. Derclaye, T. J. Foster, J. A. Geoghegan, Y. F. Dufrêne, Forces between *Staphylococcus aureus* and human skin. Nanoscale Horiz. 1, 298–303 (2016). 32260649 10.1039/c6nh00057f

[48] A. Viljoen, M. Mathelié-Guinlet, A. Ray, N. Strohmeyer, Y. J. Oh, P. Hinterdorfer, D. J. Müller, D. Alsteens, Y. F. Dufrêne, Force spectroscopy of single cells using atomic force microscopy. Nat. Rev. Methods Primers 1, 63 (2021).

[49] K. Ponnuraj, M. G. Bowden, S. Davis, S. Gurusiddappa, D. Moore, D. Choe, Y. Xu, M. Hook, S. V. Narayana, A “dock, lock, and latch” structural model for a staphylococcal adhesin binding to fibrinogen. Cell 115, 217–228 (2003). 14567919 10.1016/s0092-8674(03)00809-2

[50] X. Zhang, M. Wu, W. Zhuo, J. Gu, S. Zhang, J. Ge, M. Yang, Crystal structures of bbp from *Staphylococcus aureus* reveal the ligand binding mechanism with fibrinogen α. Protein Cell 6, 757–766 (2015). 26349459 10.1007/s13238-015-0205-x PMC4598324

[51] Y. Zhang, M. Wu, T. Hang, C. Wang, Y. Yang, W. Pan, J. Zang, M. Zhang, X. Zhang, *Staphylococcus aureus* SdrE captures complement factor H’s C-terminus via a novel ‘close, dock, lock and latch’mechanism for complement evasion. Biochem. J. 474, 1619–1631 (2017). 28258151 10.1042/BCJ20170085 PMC5415847

[52] V. K. Ganesh, J. J. Rivera, E. Smeds, Y. P. Ko, M. G. Bowden, E. R. Wann, S. Gurusiddappa, J. R. Fitzgerald, M. Höök, A structural model of the *Staphylococcus aureus* ClfA–fibrinogen interaction opens new avenues for the design of anti-staphylococcal therapeutics. PLOS Pathog. 4, e1000226 (2008). 19043557 10.1371/journal.ppat.1000226 PMC2582960

[53] H. Xiang, Y. Feng, J. Wang, B. Liu, Y. Chen, L. Liu, X. Deng, M. Yang, Crystal structures reveal the multi-ligand binding mechanism of *Staphylococcus aureus* ClfB. PLOS Pathog. 8, e1002751 (2012). 22719251 10.1371/journal.ppat.1002751 PMC3375286

[54] D. O’Connell, Dock, lock and latch. Nat. Rev. Microbiol. 1, 171 (2003).

[55] P. Herman, S. el-Kirat-Chatel, A. Beaussart, J. A. Geoghegan, T. J. Foster, Y. F. Dufrêne, The binding force of the staphylococcal adhesin SdrG is remarkably strong. Mol. Microbiol. 93, 356–368 (2014). 24898289 10.1111/mmi.12663

[56] T. Verdorfer, R. C. Bernardi, A. Meinhold, W. Ott, Z. Luthey-Schulten, M. A. Nash, H. E. Gaub, Combining in vitro and in silico single-molecule force spectroscopy to characterize and tune cellulosomal scaffoldin mechanics. J. Am. Chem. Soc. 139, 17841–17852 (2017). 29058444 10.1021/jacs.7b07574 PMC5737924

[57] N. Eswar, D. Eramian, B. Webb, M.-Y. Shen, A. Sali, “Protein structure modeling with MODELLER,” in *Structural Proteomics* (Springer, 2008), pp. 145–159. 10.1007/978-1-60327-058-8_8 18542861

[58] J. C. Phillips, D. J. Hardy, J. D. C. Maia, J. E. Stone, J. V. Ribeiro, R. C. Bernardi, R. Buch, G. Fiorin, J. Hénin, W. Jiang, R. McGreevy, M. C. R. Melo, B. K. Radak, R. D. Skeel, A. Singharoy, Y. Wang, B. Roux, A. Aksimentiev, Z. Luthey-Schulten, L. V. Kalé, K. Schulten, C. Chipot, E. Tajkhorshid, Scalable molecular dynamics on CPU and GPU architectures with NAMD. J. Chem. Phys. 153, 044130 (2020). 32752662 10.1063/5.0014475 PMC7395834

[59] J. V. Ribeiro, R. C. Bernardi, T. Rudack, J. E. Stone, J. C. Phillips, P. L. Freddolino, K. Schulten, QwikMD—Integrative molecular dynamics toolkit for novices and experts. Sci. Rep. 6, 26536 (2016). 27216779 10.1038/srep26536 PMC4877583

[60] I. Massova, P. A. Kollman, Combined molecular mechanical and continuum solvent approach (MM-PBSA/GBSA) to predict ligand binding. Perspect. Drug Discov. Des. 18, 113–135 (2000).

[61] P. A. Kollman, I. Massova, C. Reyes, B. Kuhn, S. Huo, L. Chong, M. Lee, T. Lee, Y. Duan, W. Wang, O. Donini, P. Cieplak, J. Srinivasan, D. A. Case, T. E. Cheatham III, Calculating structures and free energies of complex molecules: Combining molecular mechanics and continuum models. Acc. Chem. Res. 33, 889–897 (2000). 11123888 10.1021/ar000033j

[62] P. S. Gomes, D. E. Gomes, R. C. Bernardi, Protein structure prediction in the era of AI: Challenges and limitations when applying to in silico force spectroscopy. Front. Bioinform. 2, 983306 (2022). 36304287 10.3389/fbinf.2022.983306 PMC9580946

[63] R. Mirzaei, P. Goodarzi, M. Asadi, A. Soltani, H. Aljanabi, A. S. Jeda, S. Dashtbin, S. Jalalifar, R. Mohammadzadeh, A. Teimoori, K. Tari, M. Salari, S. Ghiasvand, S. Kazemi, R. Yousefimashouf, H. Keyvani, S. Karampoor, Bacterial co-infections with SARS-CoV-2. IUBMB Life 72, 2097–2111 (2020). 32770825 10.1002/iub.2356 PMC7436231

[64] M. Rief, M. Gautel, F. Oesterhelt, J. M. Fernandez, H. E. Gaub, Reversible unfolding of individual titin immunoglobulin domains by AFM. Science 276, 1109–1112 (1997). 9148804 10.1126/science.276.5315.1109

[65] C. Formosa-Dague, P. Speziale, T. J. Foster, J. A. Geoghegan, Y. F. Dufrêne, Zinc-dependent mechanical properties of *Staphylococcus aureus* biofilm-forming surface protein SasG. Proc. Natl. Acad. Sci. U.S.A. 113, 410–415 (2016). 26715750 10.1073/pnas.1519265113 PMC4720321

[66] C. Wang, C. Chantraine, A. Viljoen, A. B. Herr, P. D. Fey, A. R. Horswill, M. Mathelié-Guinlet, Y. F. Dufrêne, The staphylococcal biofilm protein aap mediates cell–cell adhesion through mechanically distinct homophilic and lectin interactions. PNAS Nexus 1, pgac278 (2022). 36712378 10.1093/pnasnexus/pgac278 PMC9802226

[67] L. F. Milles, E. M. Unterauer, T. Nicolaus, H. E. Gaub, Calcium stabilizes the strongest protein fold. Nat. Commun. 9, 4764 (2018). 30420680 10.1038/s41467-018-07145-6 PMC6232131

[68] J. Jumper, R. Evans, A. Pritzel, T. Green, M. Figurnov, O. Ronneberger, K. Tunyasuvunakool, R. Bates, A. Žídek, A. Potapenko, A. Bridgland, C. Meyer, S. A. A. Kohl, A. J. Ballard, A. Cowie, B. Romera-Paredes, S. Nikolov, R. Jain, J. Adler, T. Back, S. Petersen, D. Reiman, E. Clancy, M. Zielinski, M. Steinegger, M. Pacholska, T. Berghammer, S. Bodenstein, D. Silver, O. Vinyals, A. W. Senior, K. Kavukcuoglu, P. Kohli, D. Hassabis, Highly accurate protein structure prediction with AlphaFold. Nature 596, 583–589 (2021). 34265844 10.1038/s41586-021-03819-2 PMC8371605

[69] R. Alessandri, J. Barnoud, A. S. Gertsen, I. Patmanidis, A. H. de Vries, P. C. T. Souza, S. J. Marrink, Martini 3 coarse-grained force field: Small molecules. Adv. Theory Simul. 5, 2100391 (2022).

[70] M. J. Abraham, T. Murtola, R. Schulz, S. Páll, J. C. Smith, B. Hess, E. Lindahl, Gromacs: High performance molecular simulations through multi-level parallelism from laptops to supercomputers. SoftwareX 1-2, 19–25 (2015).

[71] D. J. Echelman, J. Alegre-Cebollada, C. L. Badilla, C. Chang, H. Ton-That, J. M. Fernández, CnaA domains in bacterial pili are efficient dissipaters of large mechanical shocks. Proc. Natl. Acad. Sci. U.S.A. 113, 2490–2495 (2016). 26884173 10.1073/pnas.1522946113 PMC4780631

[72] G. I. Bell, Models for the specific adhesion of cells to cells. Science 200, 618–627 (1978). 347575 10.1126/science.347575

[73] E. Evans, D. Berk, A. Leung, Detachment of agglutinin-bonded red blood cells. I. forces to rupture molecular-point attachments. Biophys. J. 59, 838–848 (1991). 2065188 10.1016/S0006-3495(91)82296-2 PMC1281249

[74] E. Evans, K. Ritchie, Dynamic strength of molecular adhesion bonds. Biophys. J. 72, 1541–1555 (1997). 9083660 10.1016/S0006-3495(97)78802-7 PMC1184350

[75] O. K. Dudko, G. Hummer, A. Szabo, Theory, analysis, and interpretation of single-molecule force spectroscopy experiments. Proc. Natl. Acad. Sci. U.S.A. 105, 15755–15760 (2008). 18852468 10.1073/pnas.0806085105 PMC2572921

[76] W. Huang, S. Le, Y. Sun, D. J. Lin, M. Yao, Y. Shi, J. Yan, Mechanical stabilization of a bacterial adhesion complex. J. Am. Chem. Soc. 144, 16808–16818 (2022). 36070862 10.1021/jacs.2c03961 PMC9501914

[77] D. E. B. Gomes, M. C. R. Melo, P. S. F. C. Gomes, R. C. Bernardi, Bridging the gap between in vitro and in silico single-molecule force spectroscopy. bioRxiv 500151 [Preprint] (2022). 10.1101/2022.07.14.500151.

[78] W. Humphrey, A. Dalke, K. Schulten, VMD: Visual molecular dynamics. J. Mol. Graph. 14, 33–38 (1996). 8744570 10.1016/0263-7855(96)00018-5

[79] M. C. Melo, R. C. Bernardi, C. De La Fuente-nunez, Z. Luthey-Schulten, Generalized correlation-based dynamical network analysis: A new high-performance approach for identifying allosteric communications in molecular dynamics trajectories. J. Chem. Phys. 153, 134104 (2020). 33032427 10.1063/5.0018980

[80] C. Schoeler, R. C. Bernardi, K. H. Malinowska, E. Durner, W. Ott, E. A. Bayer, K. Schulten, M. A. Nash, H. E. Gaub, Mapping mechanical force propagation through biomolecular complexes. Nano Lett. 15, 7370–7376 (2015). 26259544 10.1021/acs.nanolett.5b02727 PMC4721519

[81] T. Perez, W. Nelson, Cadherin adhesion: Mechanisms and molecular interactions. Handb. Exp. Pharmacol. , 3–21 (2004). 20455088 10.1007/978-3-540-68170-0_1 PMC3368609

[82] S. A. Kim, C.-Y. Tai, L.-P. Mok, E. A. Mosser, E. M. Schuman, Calcium-dependent dynamics of cadherin interactions at cell–cell junctions. Proc. Natl. Acad. Sci. U.S.A. 108, 9857–9862 (2011). 21613566 10.1073/pnas.1019003108 PMC3116393

[83] C. Schoeler, K. H. Malinowska, R. C. Bernardi, L. F. Milles, M. A. Jobst, E. Durner, W. Ott, D. B. Fried, E. A. Bayer, K. Schulten, H. E. Gaub, M. A. Nash, Ultrastable cellulosome-adhesion complex tightens under load. Nat. Commun. 5, 5635 (2014). 25482395 10.1038/ncomms6635 PMC4266597

[84] Z. Liu, H. Liu, A. M. Vera, R. C. Bernardi, P. Tinnefeld, M. A. Nash, High force catch bond mechanism of bacterial adhesion in the human gut. Nat. Commun. 11, 4321 (2020). 32859904 10.1038/s41467-020-18063-x PMC7456326

[85] M. Sotomayor, K. Schulten, The allosteric role of the Ca^2+^ switch in adhesion and elasticity of C-cadherin. Biophys. J. 94, 4621–4633 (2008). 18326636 10.1529/biophysj.107.125591 PMC2397358

[86] J. Franz, M. Beutel, K. Gevers, A. Kramer, J. P. Thyssen, S. Kezic, C. Riethmüller, Nanoscale alterations of corneocytes indicate skin disease. Skin Res. Technol. 22, 174–180 (2016). 26100642 10.1111/srt.12247

[87] S. E. Lee, S. H. Lee, Skin barrier and calcium. Ann. Dermatol. 30, 265–275 (2018). 29853739 10.5021/ad.2018.30.3.265 PMC5929942

[88] C. Riethmuller, M. A. McAleer, S. A. Koppes, R. Abdayem, J. Franz, M. Haftek, L. E. Campbell, S. F. MacCallum, W. H. I. McLean, A. D. Irvine, S. Kezic, Filaggrin breakdown products determine corneocyte conformation in patients with atopic dermatitis. J. Allergy Clin. Immunol. 136, 1573–1580.e2 (2015). 26071937 10.1016/j.jaci.2015.04.042 PMC4669308

[89] A. S. Évora, M. J. Adams, S. A. Johnson, Z. Zhang, Corneocytes: Relationship between structural and biomechanical properties. Skin Pharmacol. Physiol. 34, 146–161 (2021). 33780956 10.1159/000513054

[90] C. Riethmüller, Assessing the skin barrier via corneocyte morphometry. Exp. Dermatol. 27, 923–930 (2018). 30019542 10.1111/exd.13741

[91] C. Feuillie, P. Vitry, M. A. M. Aleer, S. Kezic, A. D. Irvine, J. A. Geoghegan, Y. F. Dufrêne, Adhesion of *Staphylococcus aureus* to corneocytes from atopic dermatitis patients is controlled by natural moisturizing factor levels. mBio 9, e01184-18 (2018). 30108169 10.1128/mBio.01184-18 PMC6094479

[92] J. W. Smith, R. S. Piotrowicz, D. Mathis, A mechanism for divalent cation regulation of beta 3-integrins. J. Biol. Chem. 269, 960–967 (1994). 7507113

[93] D. P. O’Connell, T. Nanavaty, D. McDevitt, S. Gurusiddappa, M. Höök, T. J. Foster, The fibrinogen-binding MSCRAMM (clumping factor) of *Staphylococcus aureus* has a Ca^2+^-dependent inhibitory site. J. Biol. Chem. 273, 6821–6829 (1998). 9506984 10.1074/jbc.273.12.6821

[94] M. M. King, B. B. Kayastha, M. J. Franklin, M. A. Patrauchan, Calcium regulation of bacterial virulence. Adv. Exp. Med. Biol. 1131, 827–855 (2020). 31646536 10.1007/978-3-030-12457-1_33 PMC7473484

[95] E. Josefsson, O. Hartford, L. O’Brien, J. M. Patti, T. Foster, Protection against experimental *Staphylococcus aureus* arthritis by vaccination with clumping factor a, a novel virulence determinant. J Infect Dis 184, 1572–1580 (2001). 11740733 10.1086/324430

[96] J. M. Patti, A humanized monoclonal antibody targeting *Staphylococcus aureus*. Vaccine 22, S39–S43 (2004). 15576200 10.1016/j.vaccine.2004.08.015

[97] J. J. Weems Jr., J. P. Steinberg, S. Filler, J. W. Baddley, G. R. Corey, P. Sampathkumar, L. Winston, J. F. John, C. J. Kubin, R. Talwani, T. Moore, J. M. Patti, S. Hetherington, M. Texter, E. Wenzel, V. A. Kelley, V. G. Fowler Jr., Phase ii, randomized, double-blind, multicenter study comparing the safety and pharmacokinetics of tefibazumab to placebo for treatment of *Staphylococcus aureus* bacteremia. Antimicrob. Agents Chemother. 50, 2751–2755 (2006). 16870768 10.1128/AAC.00096-06 PMC1538656

[98] L. O. Brien, S. W. Kerrigan, G. Kaw, M. Hogan, J. Penadés, D. Litt, D. J. Fitzgerald, T. J. Foster, D. Cox, Multiple mechanisms for the activation of human platelet aggregation by *Staphylococcus aureus*: Roles for the clumping factors ClfA and ClfB, the serine–aspartate repeat protein SdrE and protein A. Mol. Microbiol. 44, 1033 (2002). 12010496 10.1046/j.1365-2958.2002.02935.x

[99] O. M. Fleury, M. A. Mc Aleer, C. Feuillie, C. Formosa-Dague, E. Sansevere, D. E. Bennett, A. M. Towell, W. H. I. Mc Lean, S. Kezic, D. A. Robinson, P. G. Fallon, T. J. Foster, Y. F. Dufrêne, A. D. Irvine, J. A. Geoghegan, Clumping factor b promotes adherence of *Staphylococcus aureus* to corneocytes in atopic dermatitis. Infect. Immun. 85, e00994-16 (2017). 28373353 10.1128/IAI.00994-16 PMC5442637

[100] A. Ebner, P. Hinterdorfer, H. J. Gruber, Comparison of different aminofunctionalization strategies for attachment of single antibodies to AFM cantilevers. Ultramicroscopy 107, 922–927 (2007). 17560033 10.1016/j.ultramic.2007.02.035

[101] L. Wildling, C. Rankl, T. Haselgrübler, H. J. Gruber, M. Holy, A. H. Newman, M. F. Zou, R. Zhu, M. Freissmuth, H. H. Sitte, P. Hinterdorfer, Probing binding pocket of serotonin transporter by single molecular force spectroscopy on living cells. J. Biol. Chem. 287, 105–113 (2012). 22033932 10.1074/jbc.M111.304873 PMC3249061

[102] A. C. Dumitru, R. N. V. K. Deepak, H. Liu, M. Koehler, C. Zhang, H. Fan, D. Alsteens, Submolecular probing of the complement C5a receptor–ligand binding reveals a cooperative two-site binding mechanism. Commun. Biol. 3, 786 (2020). 33339958 10.1038/s42003-020-01518-8 PMC7749166

[103] UniProt Consortium, UniProt: A worldwide hub of protein knowledge. Nucleic Acids Res. 47, D506–D515 (2019). 30395287 10.1093/nar/gky1049 PMC6323992

[104] P. Cramer, AlphaFold2 and the future of structural biology. Nat. Struct. Mol. Biol. 28, 704–705 (2021). 34376855 10.1038/s41594-021-00650-1

[105] D. A. Case, T. E. Cheatham III, T. Darden, H. Gohlke, R. Luo, K. M. Merz Jr., A. Onufriev, C. Simmerling, B. Wang, R. J. Woods, The amber biomolecular simulation programs. J. Comput. Chem. 26, 1668–1688 (2005). 16200636 10.1002/jcc.20290 PMC1989667

[106] V. Mariani, M. Biasini, A. Barbato, T. Schwede, LDDT: A local superposition-free score for comparing protein structures and models using distance difference tests. Bioinformatics 29, 2722–2728 (2013). 23986568 10.1093/bioinformatics/btt473 PMC3799472

[107] A. Drozdetskiy, C. Cole, J. Procter, G. J. Barton, JPred4: A protein secondary structure prediction server. Nucleic Acids Res. 43, W389–W394 (2015). 25883141 10.1093/nar/gkv332 PMC4489285

[108] H. M. Berman, J. Westbrook, Z. Feng, G. Gilliland, T. N. Bhat, H. Weissig, I. N. Shindyalov, P. E. Bourne, The protein data bank. Nucleic Acids Res. 28, 235–242 (2000). 10592235 10.1093/nar/28.1.235 PMC102472

[109] R. C. Edgar, Muscle: Multiple sequence alignment with high accuracy and high throughput. Nucleic Acids Res. 32, 1792–1797 (2004). 15034147 10.1093/nar/gkh340 PMC390337

[110] M. Johnson, I. Zaretskaya, Y. Raytselis, Y. Merezhuk, S. McGinnis, T. L. Madden, NCBI BLAST: A better web interface. Nucleic Acids Res. 36, W5–W9 (2008). 18440982 10.1093/nar/gkn201 PMC2447716

[111] M.-Y. Shen, A. Sali, Statistical potential for assessment and prediction of protein structures. Protein Sci. 15, 2507 (2006). 17075131 10.1110/ps.062416606 PMC2242414

[112] O. J. Harrison, J. Brasch, G. Lasso, P. S. Katsamba, G. Ahlsen, B. Honig, L. Shapiro, Structural basis of adhesive binding by desmocollins and desmogleins. Proc. Natl. Acad. Sci. U.S.A. 113, 7160–7165 (2016). 27298358 10.1073/pnas.1606272113 PMC4932976

[113] R. B. Best, X. Zhu, J. Shim, P. E. M. Lopes, J. Mittal, M. Feig, A. D. MacKerell Jr., Optimization of the additive charmm all-atom protein force field targeting improved sampling of the backbone ϕ, ψ and side-chain χ_1_ and χ_2_ dihedral angles. J. Chem. Theory Comput. 8, 3257–3273 (2012). 23341755 10.1021/ct300400x PMC3549273

[114] W. L. Jorgensen, J. Chandrasekhar, J. D. Madura, R. W. Impey, M. L. Klein, Comparison of simple potential functions for simulating liquid water. J. Chem. Phys. 79, 926–935 (1983).

[115] T. Darden, D. York, L. Pedersen, Particle mesh Ewald: An *N*· log (*N*) method for Ewald sums in large systems. J. Chem. Phys. 98, 10089–10092 (1993).

[116] P. C. Souza, R. Alessandri, J. Barnoud, S. Thallmair, I. Faustino, F. Grünewald, I. Patmanidis, H. Abdizadeh, B. M. H. Bruininks, T. A. Wassenaar, P. C. Kroon, J. Melcr, V. Nieto, V. Corradi, H. M. Khan, J. Domański, M. Javanainen, H. Martinez-Seara, N. Reuter, R. B. Best, I. Vattulainen, L. Monticelli, X. Periole, D. P. Tieleman, A. H. de Vries, S. J. Marrink, Martini 3: A general purpose force field for coarse-grained molecular dynamics. Nat. Methods 18, 382–388 (2021). 33782607 10.1038/s41592-021-01098-3 PMC12554258

[117] P. C. Kroon, T. A. Wassenaar, J. Barnoud, S.-J. Marrink, New automated and high-throughput tools for the Martini forcefield. Biophys. J. 114, 676A–677A (2018).

[118] K. Wołek, À. Gómez-Sicilia, M. Cieplak, Determination of contact maps in proteins: A combination of structural and chemical approaches. J. Chem. Phys. 143, 243105 (2015). 26723590 10.1063/1.4929599

[119] A. B. Poma, M. Cieplak, P. E. Theodorakis, Combining the Martini and structure-based coarse-grained approaches for the molecular dynamics studies of conformational transitions in proteins. J. Chem. Theory Comput. 13, 1366–1374 (2017). 28195464 10.1021/acs.jctc.6b00986

[120] G. Bussi, D. Donadio, M. Parrinello, Canonical sampling through velocity rescaling. J. Chem. Phys. 126, 014101 (2007). 17212484 10.1063/1.2408420

[121] D. H. De Jong, S. Baoukina, H. I. Ingólfsson, S. J. Marrink, Martini straight: Boosting performance using a shorter cutoff and GPUs. Comput. Phys. Commun. 199, 1–7 (2016).

[122] I. G. Tironi, R. Sperb, P. E. Smith, W. F. van Gunsteren, A generalized reaction field method for molecular dynamics simulations. J. Chem. Phys. 102, 5451–5459 (1995).

[123] L. Verlet, Computer “experiments” on classical fluids. I. thermodynamical properties of lennard-jones molecules. Phys. Rev. 159, 98–103 (1967).

[124] B. Hess, H. Bekker, H. J. Berendsen, J. G. Fraaije, Lincs: A linear constraint solver for molecular simulations. J. Comput. Chem. 18, 1463–1472 (1997).

[125] O. K. Dudko, G. Hummer, A. Szabo, Intrinsic rates and activation free energies from single-molecule pulling experiments. Phys. Rev. Lett. 96, 108101 (2006). 16605793 10.1103/PhysRevLett.96.108101

[126] D. E. Gomes, B. Yang, R. Vanella, M. A. Nash, R. C. Bernardi, Integrating dynamic network analysis with AI for enhanced epitope prediction in PD-L1: Affibody interactions. J. Am. Chem. Soc. 146, 23842–23853 (2024). 39146039 10.1021/jacs.4c05869

[127] M. Spivak, J. E. Stone, J. Ribeiro, J. Saam, L. Freddolino, R. C. Bernardi, E. Tajkhorshid, VMD as a platform for interactive small molecule preparation and visualization in quantum and classical simulations. J. Chem. Inf. Model. 63, 4664–4678 (2023). 37506321 10.1021/acs.jcim.3c00658 PMC10516160

[128] J. D. Hunter, Matplotlib: A 2D graphics environment. Comput. Sci. Eng. 9, 90–95 (2007).

[129] W. McKinney, “Data structures for statistical computing in Python,” in *Proceedings of the 9th Python in Science Conference* (SciPy, 2010), vol. 445, pp. 51–56.

[130] M. L. Waskom, Seaborn: Statistical data visualization. J. Open Source Softw. 6, 3021 (2021).

