# Supplementary material for: Ultrastrong Staphylococcus aureus adhesion to human skin: Calcium as a key regulator of noncovalent interactions
Source: Sci Adv. 2025 Sep 3;11(36):eadu7457. doi: 10.1126/sciadv.adu7457 (PMC12407086; doi:10.1126/sciadv.adu7457)
Supplement: Supplementary file 1 — Figs. S1 to S19 Tables S1 to S3 Legend for data S1 [file sciadv.adu7457_sm.pdf]

Science Advances
AAAS

Supplementary Materials for

Ultrastrong *Staphylococcus aureus* adhesion to human skin: Calcium as a key regulator of noncovalent interactions

Constance Chantraine *et al.*

Corresponding author: Joan A. Geoghegan, j.geoghegan@bham.ac.uk; Rafael C. Bernardi, rcbernardi@auburn.edu; Yves F. Dufrêne, yves.dufrene@uclouvain.be

Sci. Adv. **11**, eadu7457 (2025)
DOI: 10.1126/sciadv.adu7457

The PDF file includes:

Figs. S1 to S19
Tables S1 to S3
Legend for data S1

Other Supplementary Material for this manuscript includes the following:

Data S1

Supplementary Figures

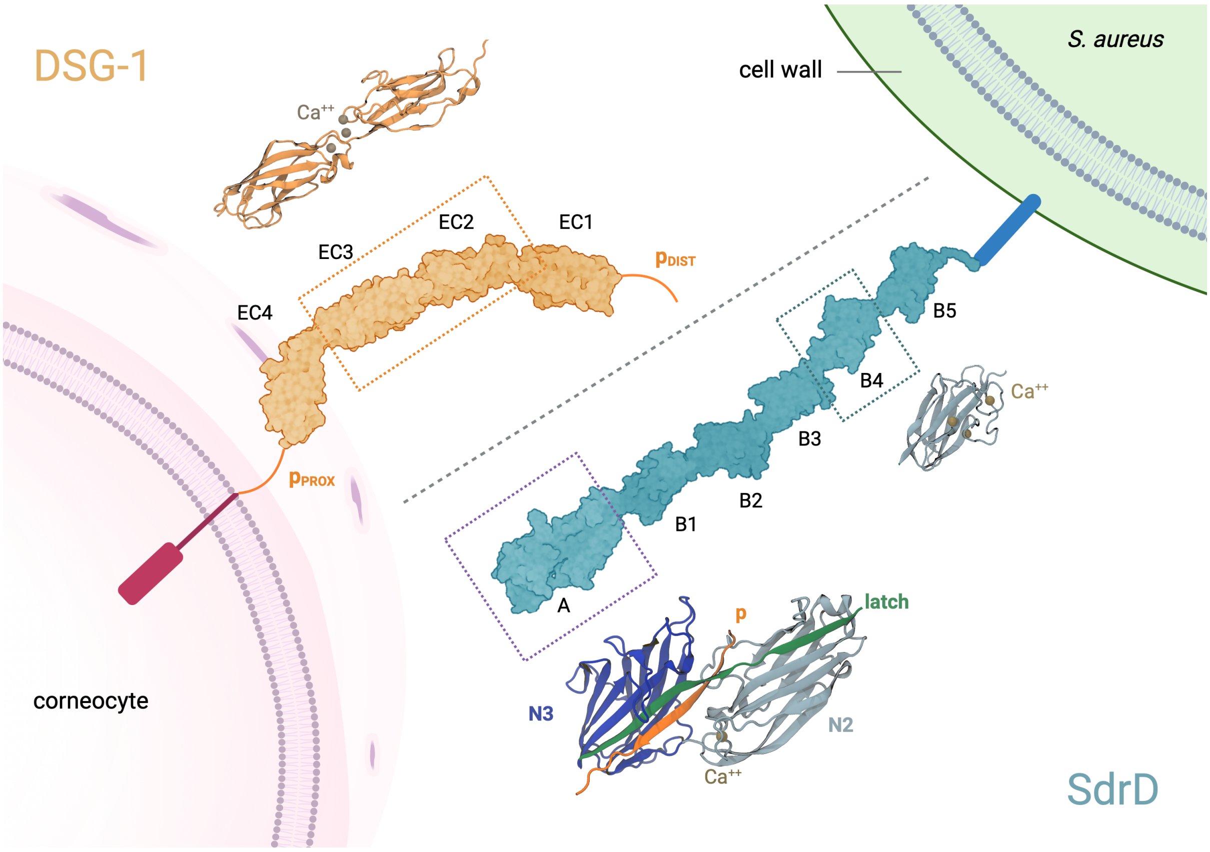

Figure S1. SdrD and DSG-1 detailed structure composition. Schematic representation for the cellular localization for both SdrD and DSG-1 proteins. The skin corneocyte is represented in pink, where DSG-1 is anchored on the cell surface and its four extracellular (EC) domains are shown in a surface representation, colored in orange. Unstructured regions that originated both p_{DIST} and p_{PROX} are indicated. Details on the tridimensional structure of each EC cadherin domain are represented as an insert, in cartoon, where we see Ca^{++} ions that stabilize the interface between adjacent EC domains. The *S. aureus* cell is colored in green, where anchored to the cell wall is SdrD, composed of its A and five consecutive B domains, colored in cyan. Details on the structure of an individual B domain is seen as an insert, with its fold stabilized by Ca^{++} ions. Another insert shows the A domain in more detail. SdrD A domain has an Ig-like fold and is divided into N2 and N3 domains, colored in dark and light blue, respectively. Between N2 and N3, the peptide (orange) binding site lies in an hydrophobic trench. Upon binding or docking of the peptide, the once unstructured latch motif (green) undergoes a conformational change that locks the peptide on the conformation that we see on the image, forming an additional β strand bound to the βE in the N2 domain. This mechanism is called "dock, lock and latch" or DLL. Created in BioRender. Bernardi, R. (2025) https://BioRender.com/mq544q7

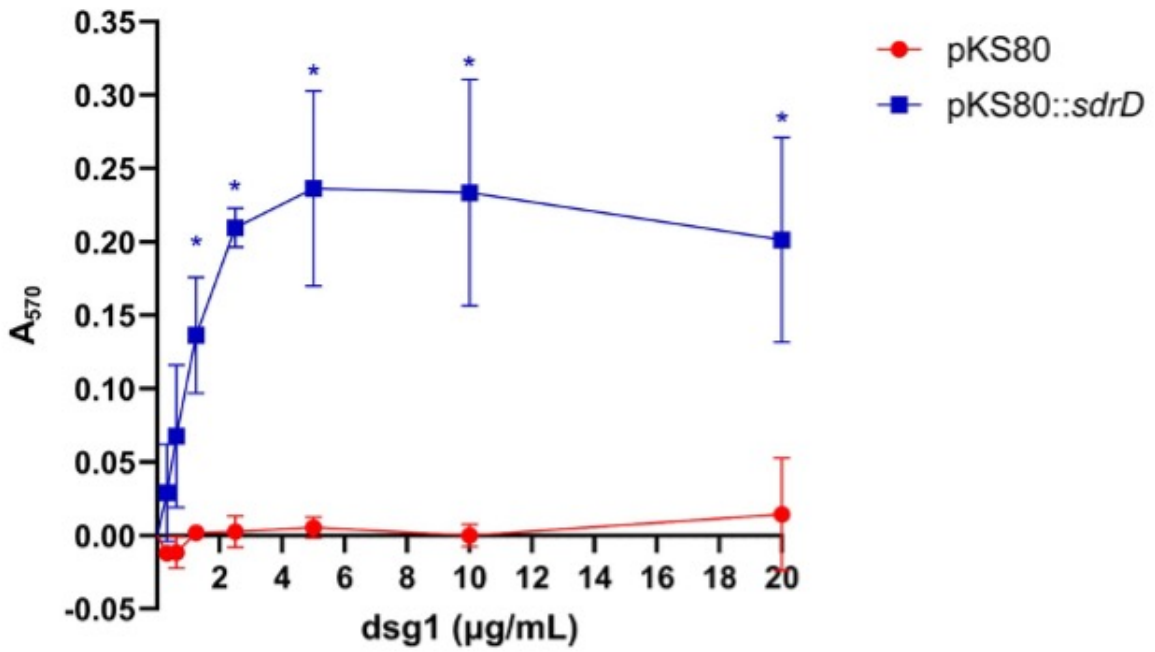

Figure S2. ***L. lactis* ectopically expresses SdrD.** *In vitro* adherence assays confirm the presence of SdrD on the surface of *L. lactis* SdrD$^{(+)}$, and its absence in SdrD$^{(-)}$. These results are based on three biological replicates and demonstrate successful surface expression of SdrD in our system and show that interactions with DSG-1 are mediated solely by SdrD in *L. lactis*.

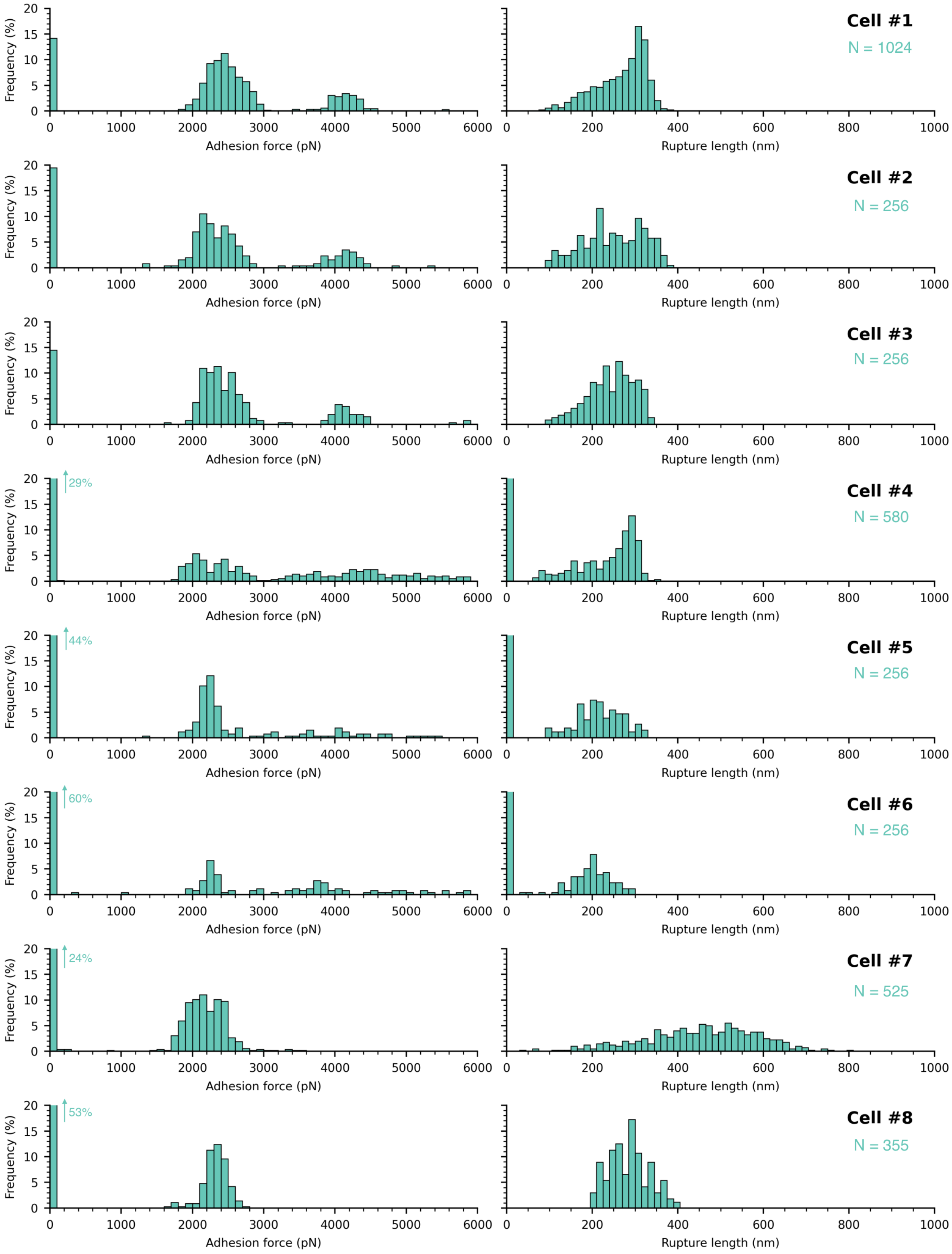

Figure S3. Single-cell force spectroscopy of the SdrD$^{(+)}$:corneocyte interaction. Adhesion force and rupture lengths histograms are obtained by recording force-distance curves in phosphate buffered saline (PBS) between bacterial probe and healthy corneocyte.

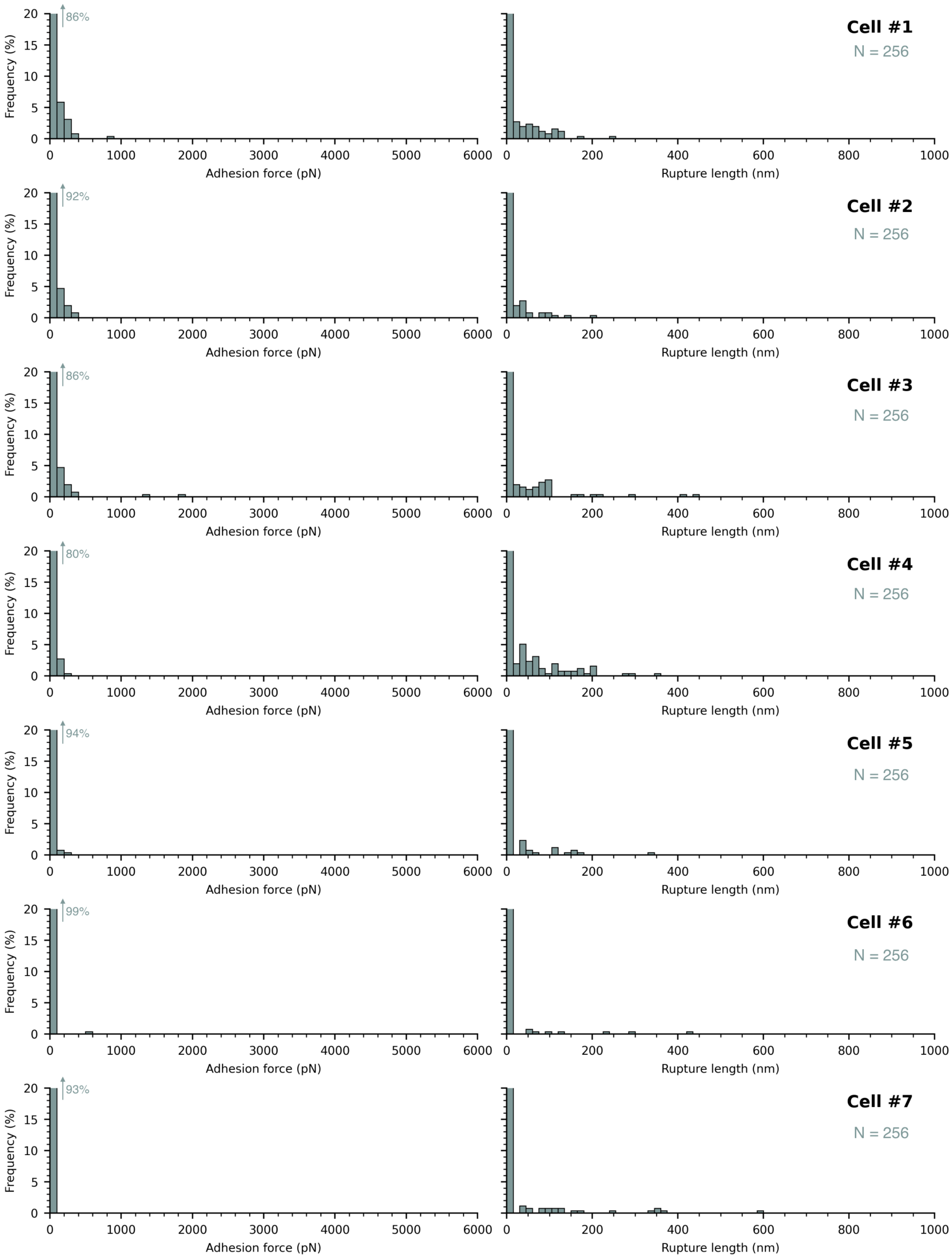

Figure S4. Single-cell force spectroscopy of the SdrD$^{(-)}$:corneocyte interaction. Adhesion force and rupture lengths histograms are obtained by recording force-distance curves in phosphate buffered saline (PBS) between bacterial probe and healthy corneocyte.

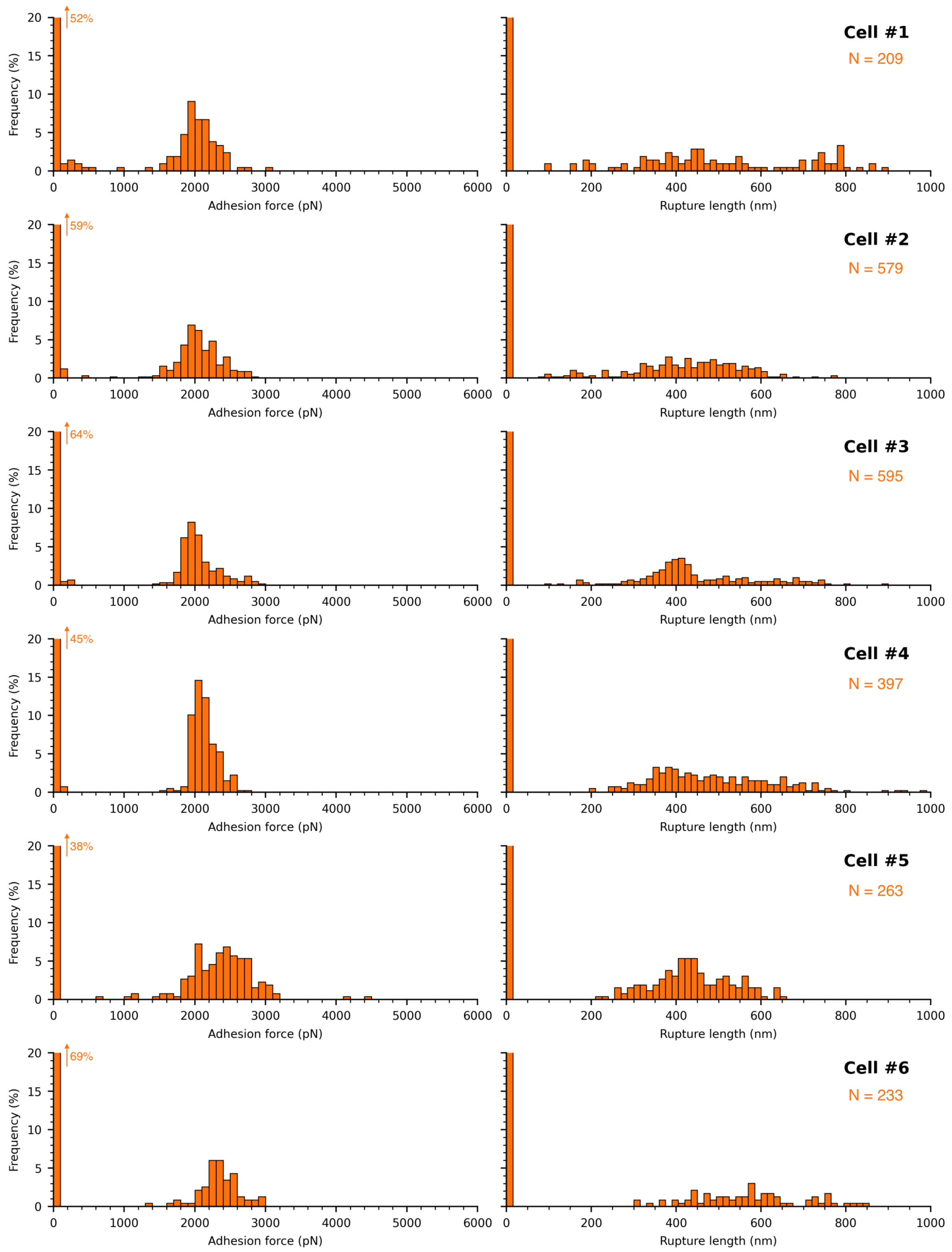

Figure S5. Single-molecule force spectroscopy of the SdrD$^{(+)}$:DSG-1 interaction. Adhesion force and rupture lengths histograms are obtained by recording force-distance curves in phosphate buffered saline (PBS) between DSG-1 modified tip and the bacteria.

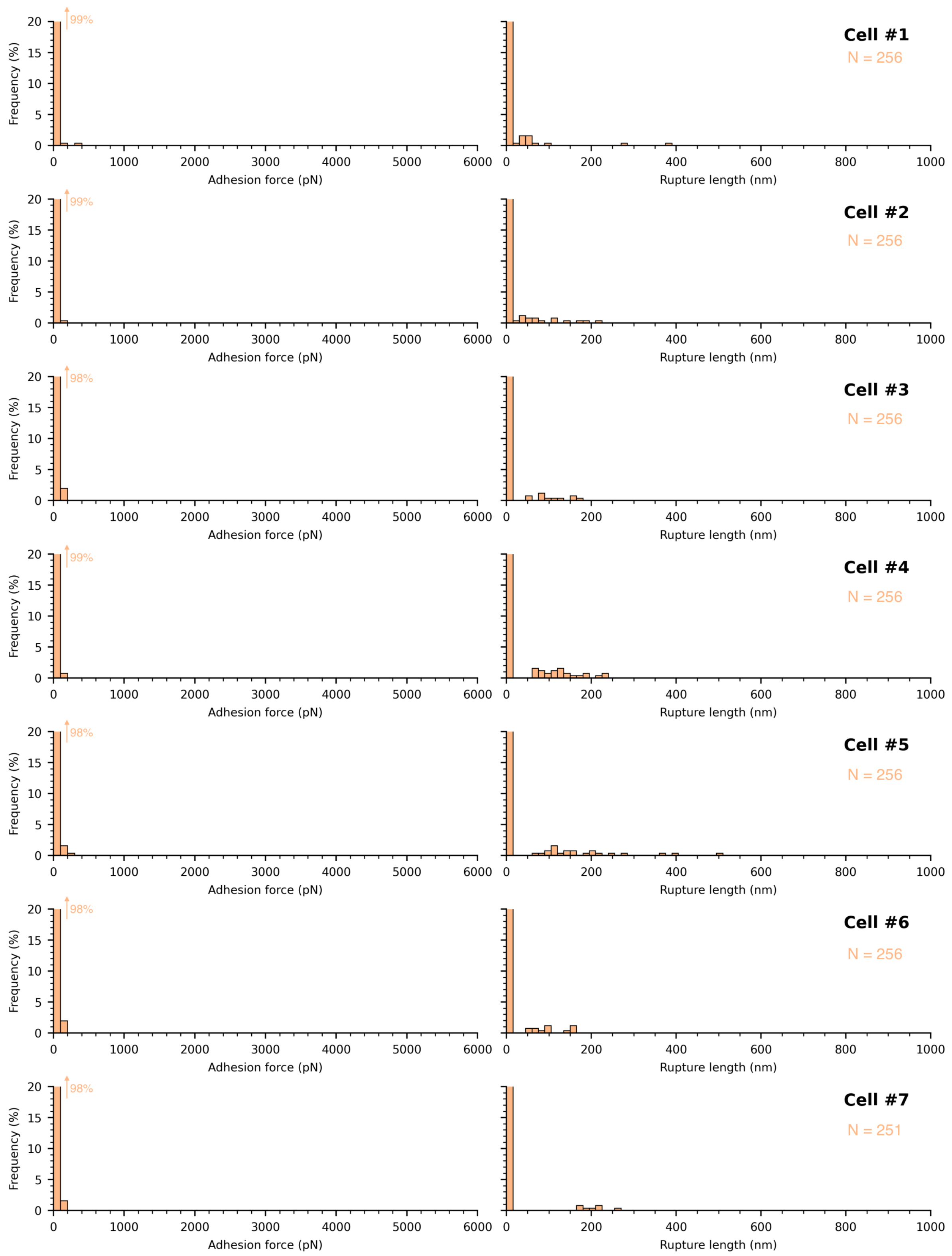

Figure S6. Single-molecule force spectroscopy of the SdrD$^{(-)}$:DSG-1 interaction. Adhesion force and rupture lengths histograms are obtained by recording force-distance curves in phosphate buffered saline (PBS) between DSG-1 modified tip and the bacteria.

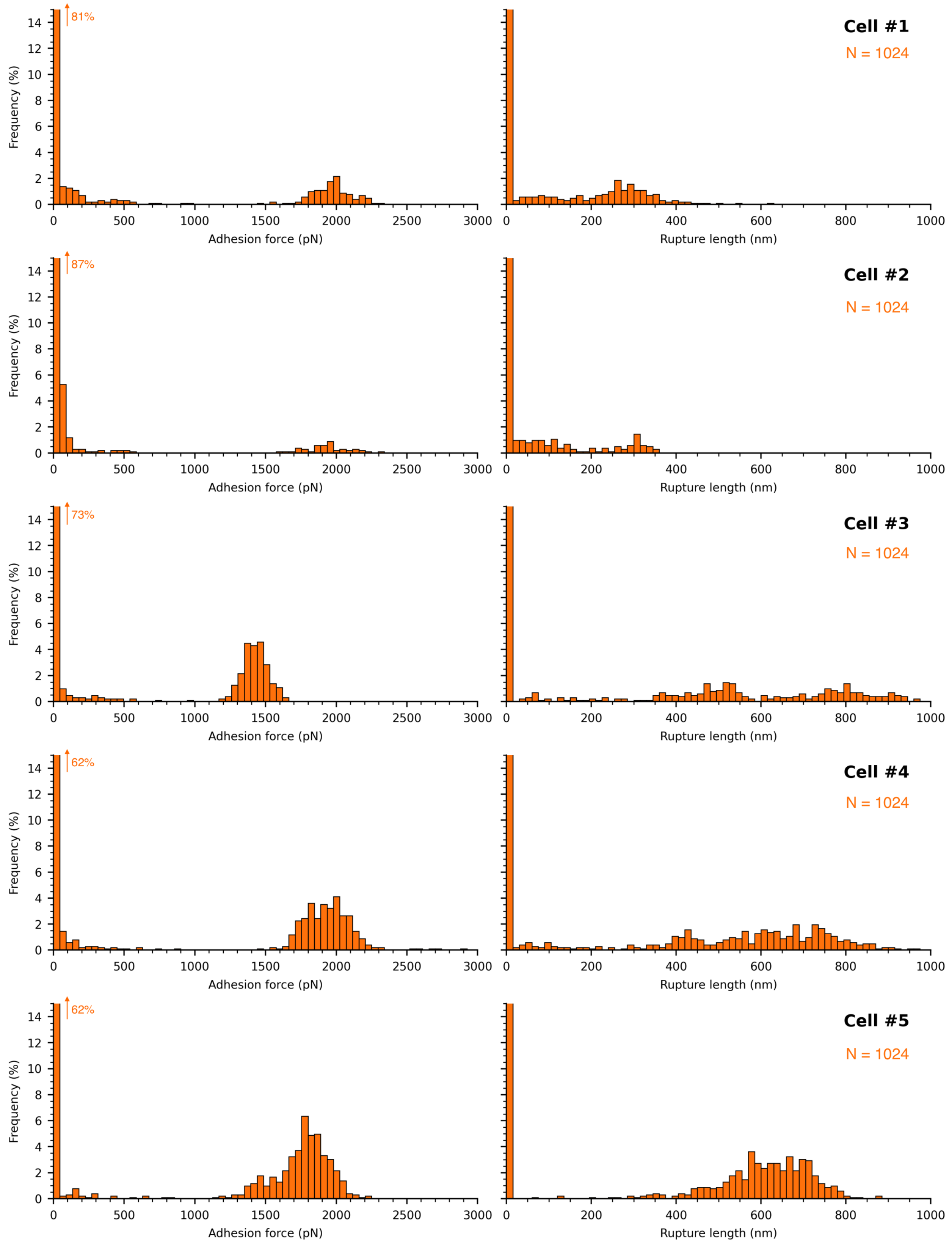

Figure S7. Single-molecule force spectroscopy of the SdrD$^{(+)}$:p$^{*}_{\mathbf{PROX}}$. Adhesion force and rupture lengths histograms are obtained by recording force-distance curves in phosphate buffered saline (PBS) between DSG-1 modified tip with p$^{*}_{\mathrm{PROX}}$ and the bacteria.

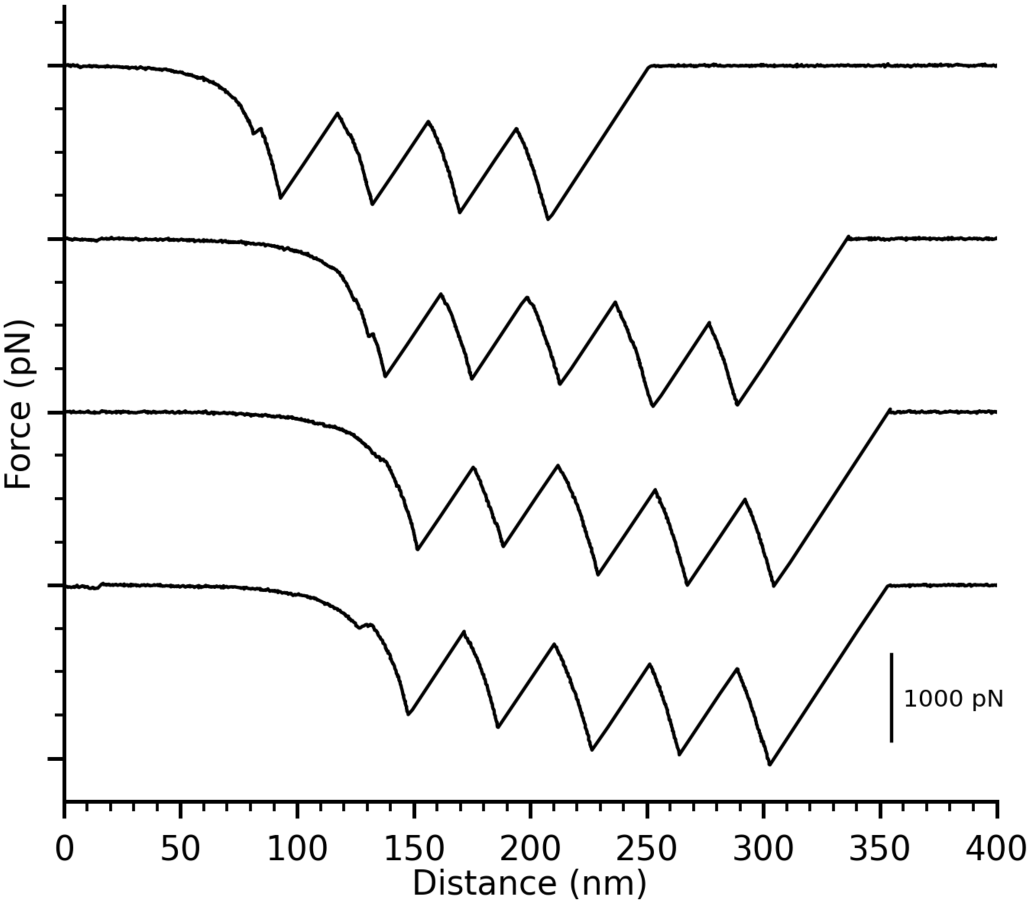

Figure S8. Sawtooth pattern for SdrD$^{(+)}$:p$^{*}_{PROX}$. Canonical sawtooth pattern detected on single-molecule force spectroscopy (SMFS) experiments for *L. lactis* SdrD$^{(+)}$ binding to p$^{*}_{PROX}$.

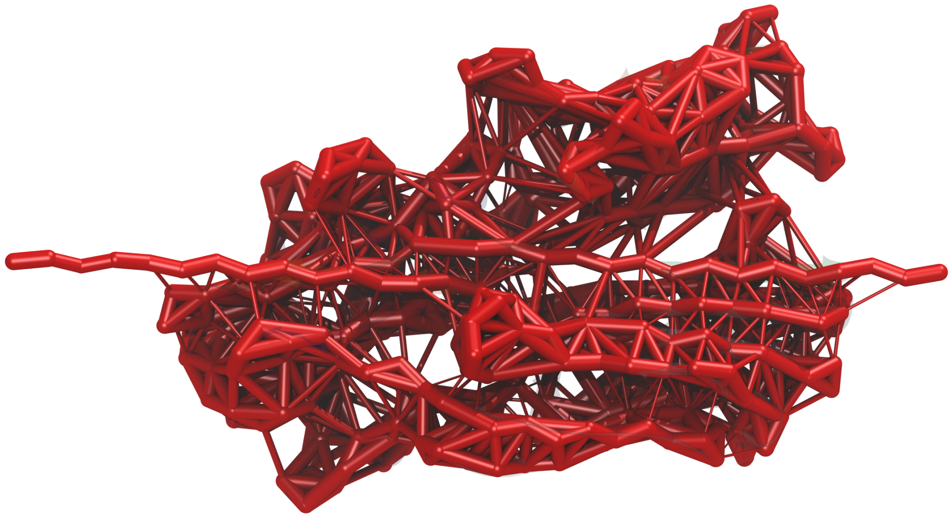

Figure S9. SdrD:DSG-1^{PPROX} dynamical network under mechanical load. The hyperstability of the protein complex can be investigated by analysing the evolution of pairwise interactions during the steered molecular dynamics (SMD) simulations [79]. The dynamical network is represented in red traces where the thickness between the nodes (amino acids) represents the correlation of motion between these residues.

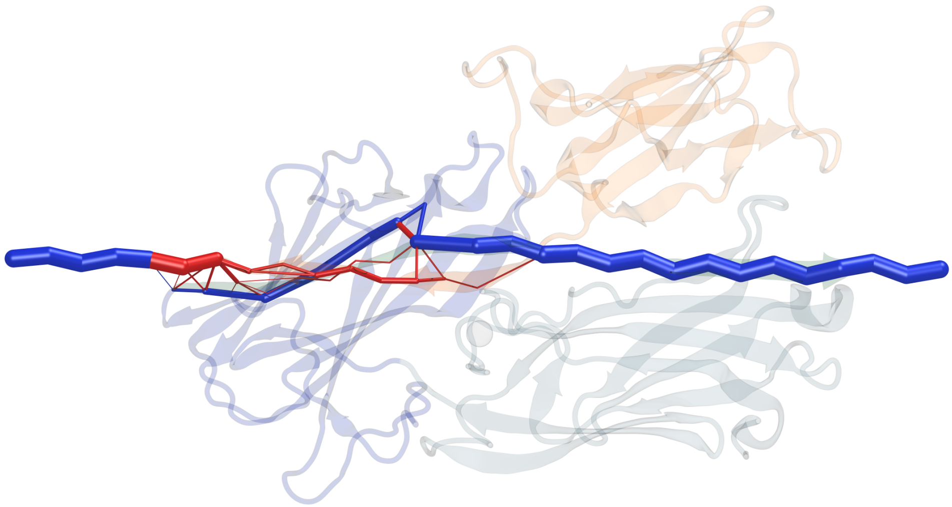

Figure S10. SdrD:DSG-1^{PPROX} force propagation pathway between anchor and pulling points. The force propagates from the latch indirectly to the peptide passing by the N3 domain of the protein. SdrD N2, N3 domains and the latch are represented in transparent cartoon, colored in dark blue, light blue and green, respectively. DSG-1 is also represented in transparency and colored in orange. The network's optimal path is colored in dark blue while the sub-optimal paths are colored in red.

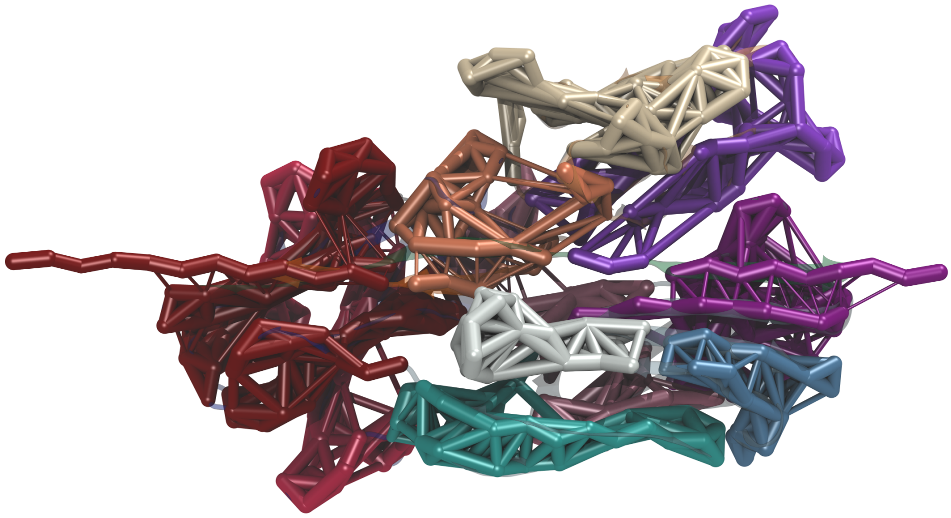

Figure S11. SdrD:DSG-1^{PPROX} network communities. The full network is colored by correlation-based communities. Communities are representations of a network into subgroups of most inter-connected residues in reference to the rest of the network. Here we observe that part of the latch is connected with the N2 domain (purple), part of extracellular domain EC4 is also grouped with the latch motif (violet) and a central community is connecting the peptide, the latch and the N3 domain (salmon).

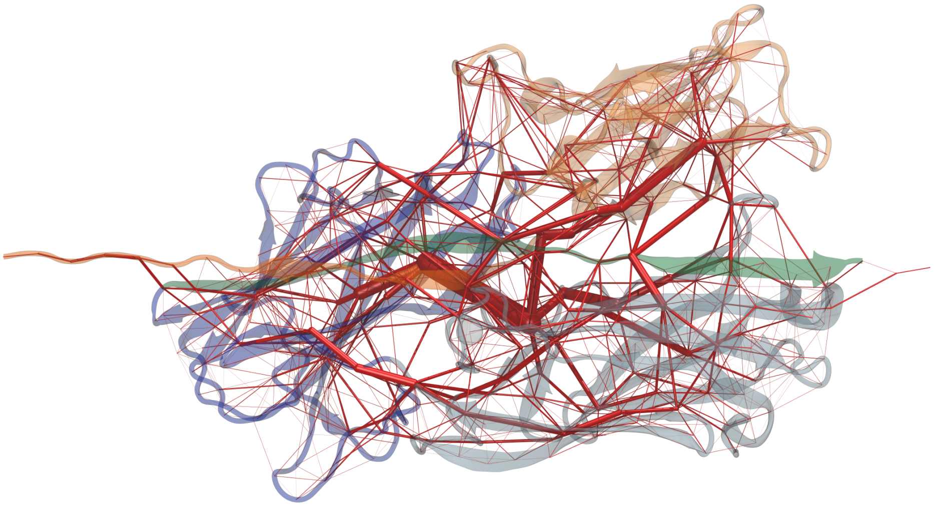

Figure S12. SdrD:DSG-1^{PPROX} betweenness network map. As in Fig.S9, the full network is represented here but the weight, or the thickness, of the network edges is given by the betweenness values. The betweenness is defined as the number of shortest paths from all vertices to all others that pass through that node, in this case, an amino acid residue. If an amino acid residue has high betweenness, it tends to be important for controlling inter-domain communication within a protein [79]. High betweenness is observed on pathways that connect extracellular domain EC4 and both N2 and N3 domains of SdrD, contributing to make the complex more rigid under high force loads.

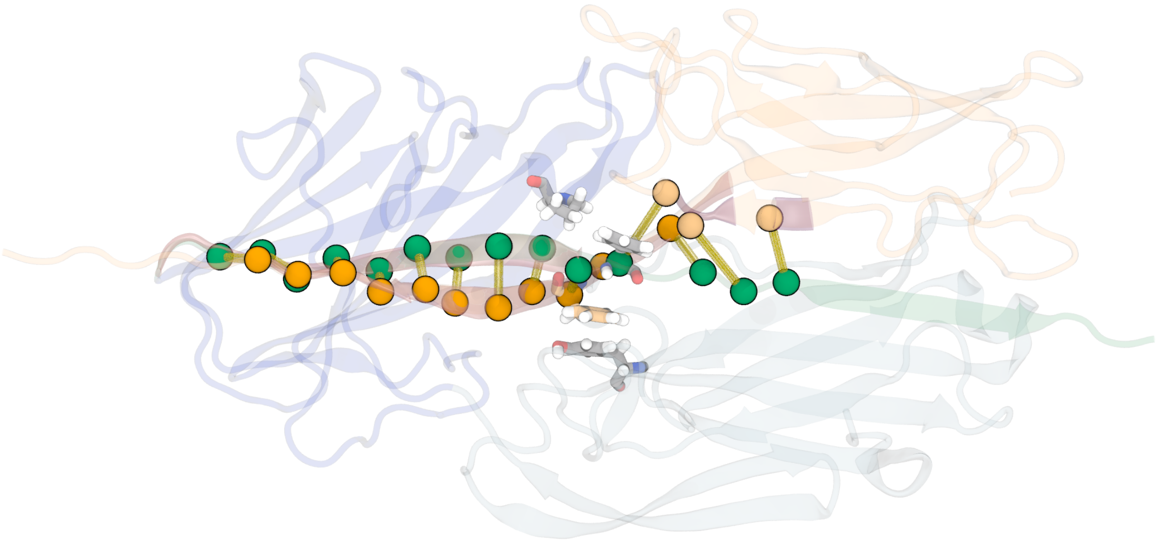

Figure S13. SdrD:DSG-1$^{p_{PROX}}$ most highly correlated pairs. SdrD:DSG-1$^{p_{PROX}}$ highly correlated residue-pairs are shown, with a focus on the protein:peptide interface. The protein complex is represented in cartoon, following the same coloring scheme used throughout the paper, with highly correlated regions colored as in **Fig. 5e**. The residues involved in π-stacking interactions are represented as sticks and colored in grey for SdrD, and orange for p_{PROX}. Residue-pairs alpha carbons are shown as van der Waals spheres with dotted yellow lines representing the correlations. The following pairs are shown: 561-391, 560-393, 559-483, 557-485, 557-394, 556-486, 555-487, 554-488, 553-489, 552-490, 551-491, 550-492, 549-493, 548-493, 547-494, following the SdrD and DSG-1 residue numbering, respectively.

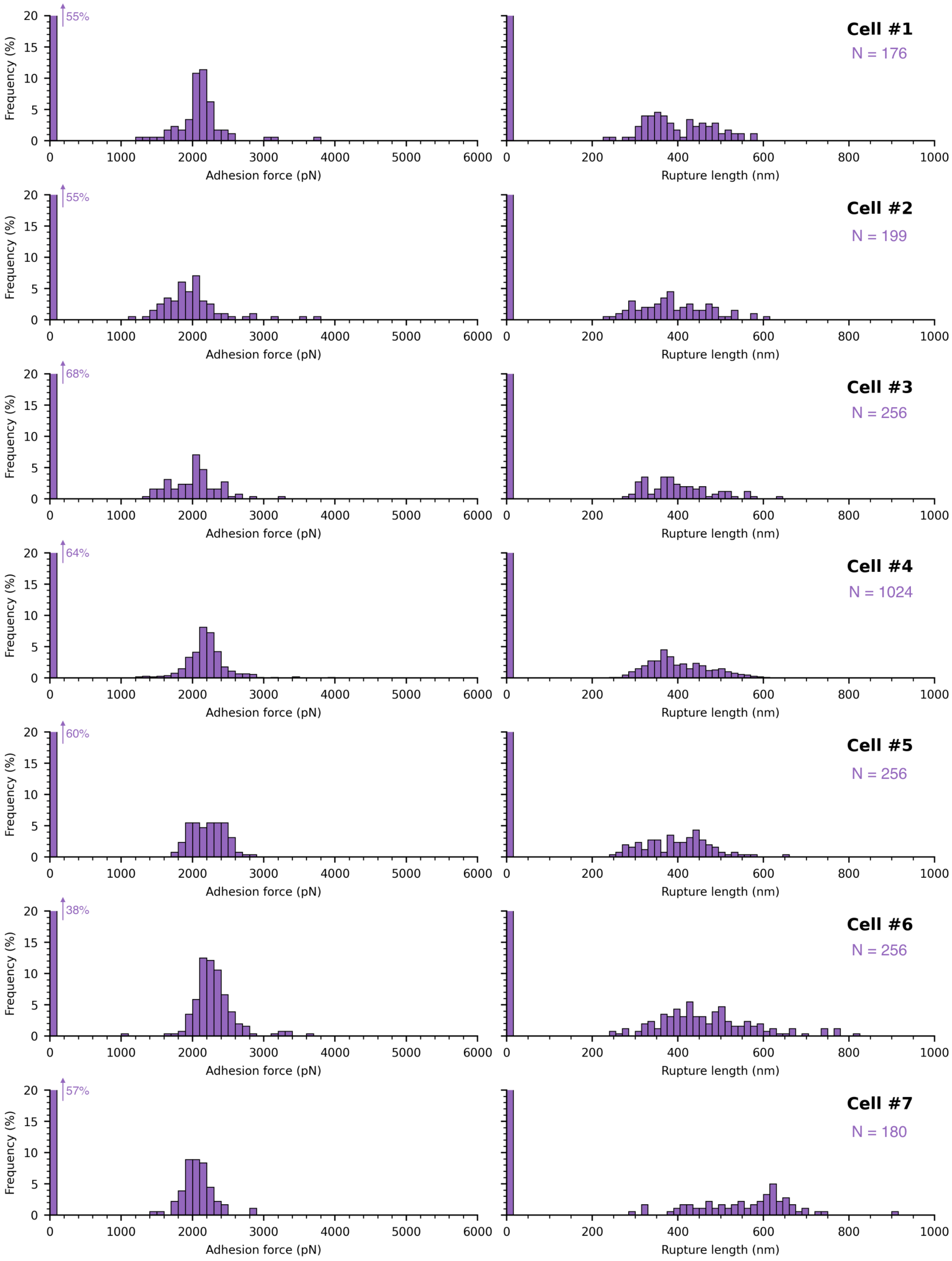

Figure S14. Single-molecule force spectroscopy of the SdrD$^{(+)}$:DSG-1 interaction in presence of 1 mM EDTA. Adhesion force and rupture lengths histograms are obtained by recording force-distance curves in phosphate buffered saline (PBS) between DSG-1 modified tip and the bacteria.

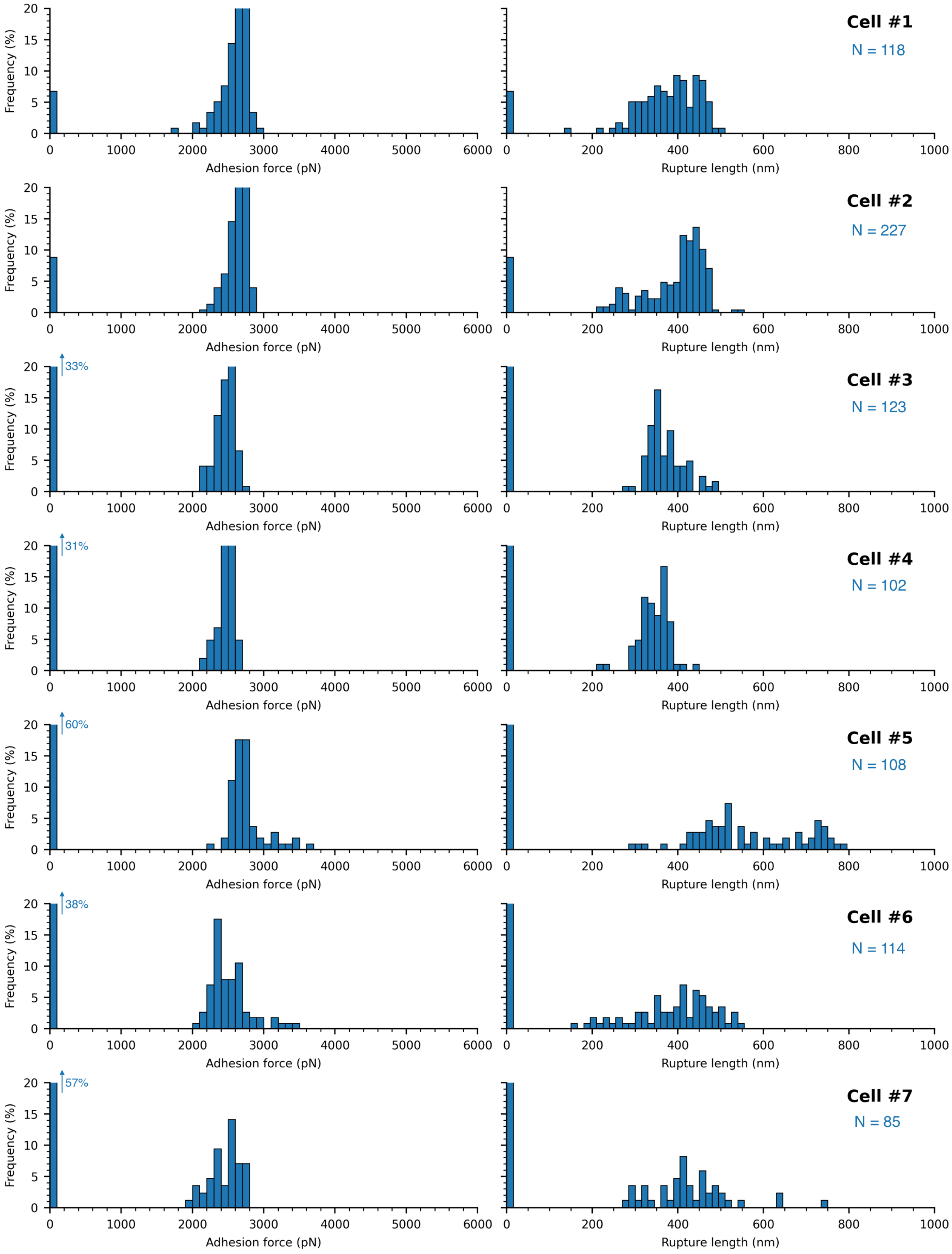

Figure S15. Single-molecule force spectroscopy of the SdrD$^{(+)}$:DSG-1 interaction in presence of 1 mM EDTA and 10 mM Ca^{++}. Adhesion force and rupture lengths histograms are obtained by recording force-distance curves in phosphate buffered saline (PBS) between DSG-1 modified tip and the bacteria.

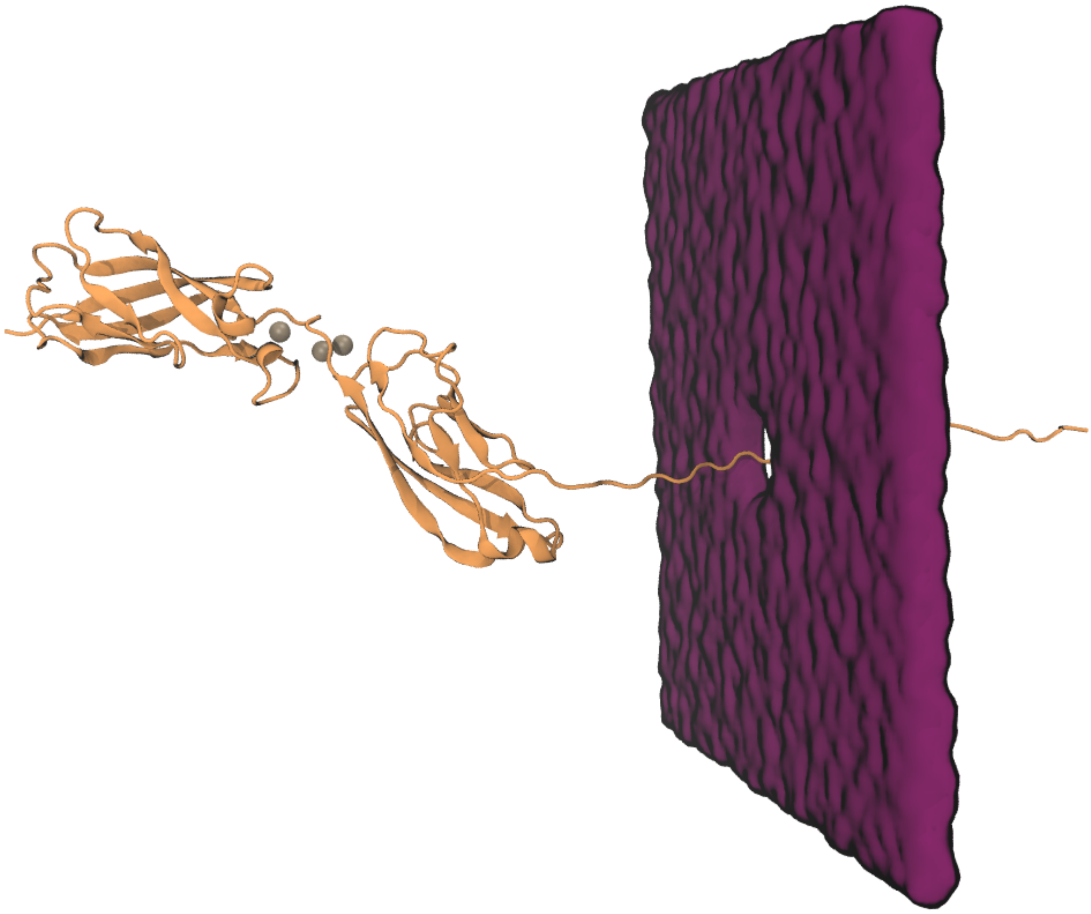

Figure S16. Probing the role of calcium on stabilizing adjacent extracellular (EC) cadherin domains. Illustration of the toy system created to investigate the role of calcium on the unfolding of EC cadherin domains upon high force. EC4 and EC3 cadherin domains are represented in cartoon, colored in orange and calcium ions are represented in spheres, colored in tan. The fixed atom nanosheet is represented in vdW surface, colored in purple. Pulling is done by the free end terminal of the unstructured p_{PROX} peptide.

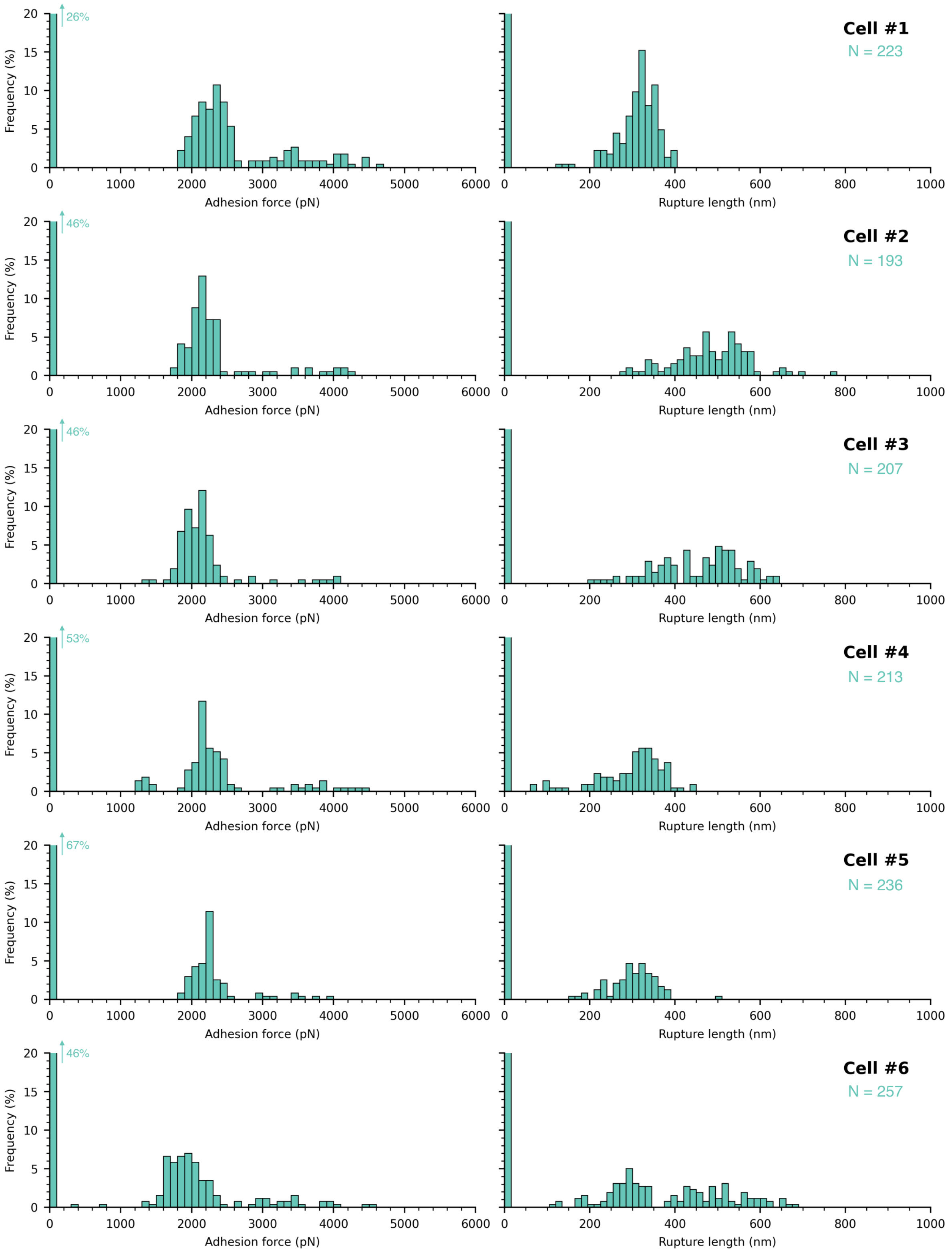

Figure S17. Single-cell force spectroscopy of the SdrD$^{(+)}$:atopic dermatitis (AD) corneocyte interaction. Adhesion force and rupture lengths histograms are obtained by recording force-distance curves in phosphate buffered saline (PBS) between bacterial probe and the AD corneocyte.

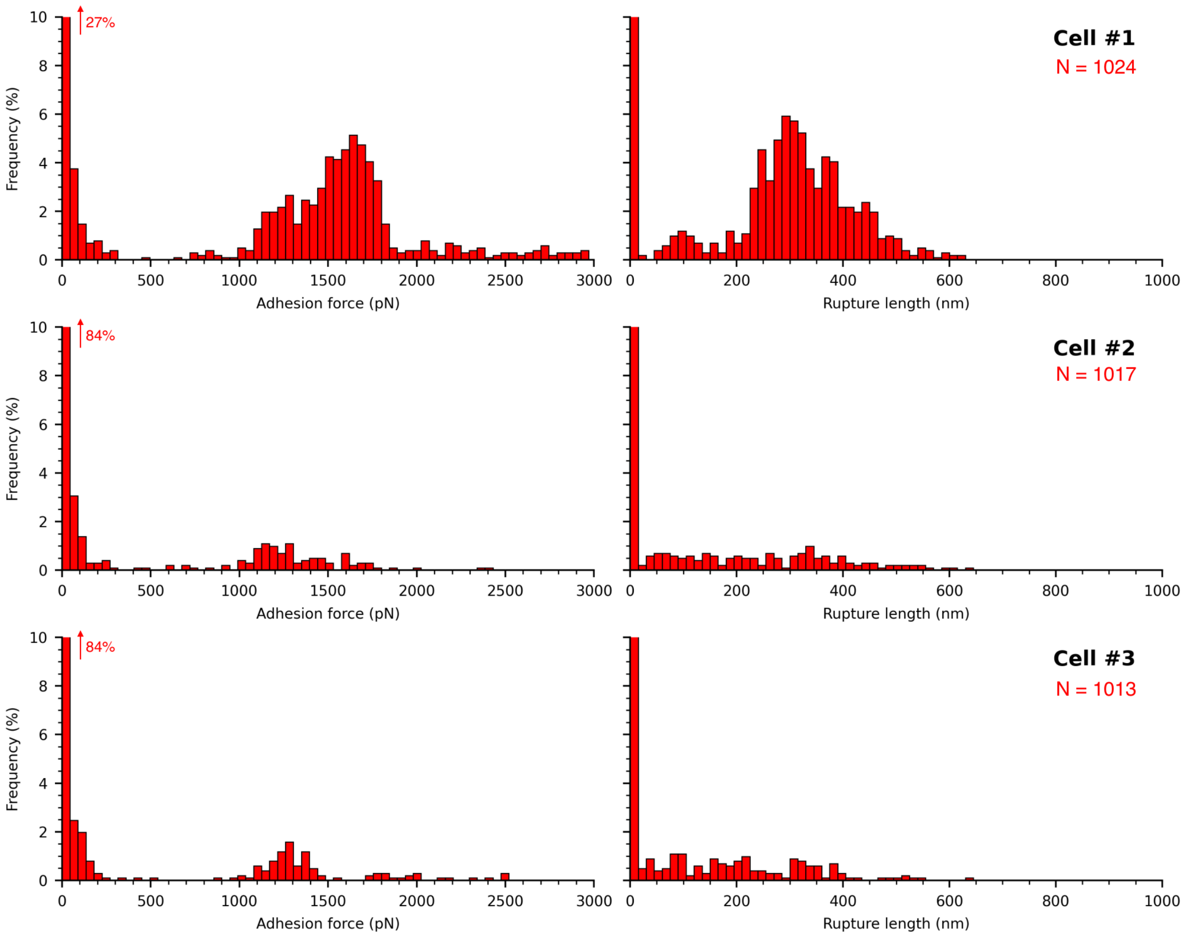

Figure S18. Single-molecule force spectroscopy of the *S. aureus* AD08:DSG-1 interaction. Adhesion force and rupture lengths histograms are obtained by recording force-distance curves in phosphate buffered saline (PBS) between bacterial probe and the atopic dermatitis (AD) corneocyte.

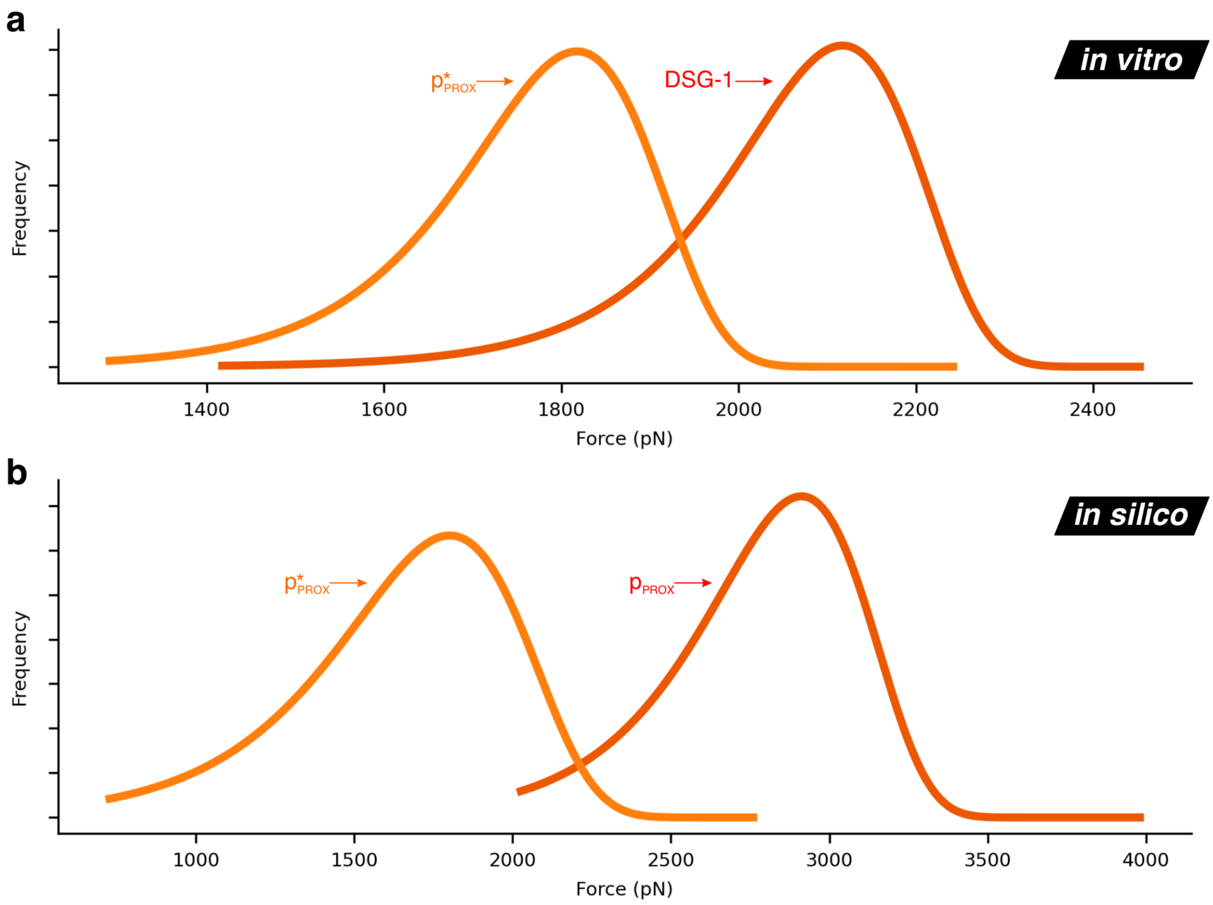

Figure S19. Force distribution curve for rupture events with and without the extracellular domain EC4. The plot shows Bell-Evans fits of rupture events observed both *in vitro* and *in silico*. a. presents experimental data, revealing a significant reduction in force resilience when comparing the full DSG-1 protein to the peptide p^*_{PROX} alone. b. confirms that this reduction is also observed *in silico*. These results highlight the essential role of cadherin domains in the modified "dock, lock and latch" mechanism. Notably, despite the decrease in rupture forces, the complex still exhibits nanoNewton-level force resilience, placing it among the strongest protein interactions reported, even in the absence of the EC4 domain.

Supplementary Tables

Table S1. Number of adhesive events estimated over single-cell and single-molecule force spectroscopy experiments.

**Experiment**	**Adhesive events** (> 0 pN)	**Total number of measurements**	**Cells**
SdrD(+):corneocyte	2721	3765	9
SdrD(-):corneocyte	215	2048	8
SdrD(+):DSG-1	1229	2580	7
SdrD(-):DSG-1	128	2043	8
SdrD(+):DSG-1 1mM EDTA	952	2347	7
SdrD(+):DSG-1 1mM EDTA 10 mM Ca^{++}	658	877	7
SdrD(+):p^*_{PROX}	2020	6139	6
SdrD(+):AD	816	1541	7
SdrD(+):AD08	1592	4055	4

Table S2. All-atom steered molecular dynamics (SMD) simulations and corresponding number of replicas for each pulling velocity.

**System**	**Pulling speed** (nm/ps)	**Replicas**	**Time** (ns)	**Total** (ns)
p_{DIST}				
$SdrD^{A\text{-}domain}$:$DSG\text{-}1^{25\text{-}42}$	5.0×10^{-04}	128	20	2,560
	5.0×10^{-05}	128	120	15,360
p_{PROX}				
$SdrD^{A\text{-}domain}$:$DSG\text{-}1^{378\text{-}500}$	5.0×10^{-03}	128	1.2	154
	5.0×10^{-04}	128	12	1,536
	5.0×10^{-05}	128	120	15,360
	5.0×10^{-06}	80	900	72,000
p^{*}_{PROX}				
$SdrD^{A\text{-}domain}$:$DSG\text{-}1^{484\text{-}497}$	5.0×10^{-05}	32	120	3,840
**Total for p_{PROX}**				89,050
**Total for all**				110,810

Table S3. Coarse-grained steered molecular dynamics (SMD) simulations and corresponding number of replicas for each pulling velocity.

**System**	**Pulling speed** (nm/ps)	**Replicas**	**Time** (ns)	**Total** (ns)
**p_{DIST}**				
SdrD$^{244\text{-}1129}$:DSG-1$^{23\text{-}500}$	2.5×10^{-04}	3	2,000	6,000
**p_{PROX}**				
SdrD$^{244\text{-}570}$:DSG-1$^{378\text{-}500}$	5.0×10^{-06}	32	500	16,000
SdrD$^{244\text{-}570}$:DSG-1$^{378\text{-}500}$	5.0×10^{-07}	32	3,000	96,000
SdrD$^{244\text{-}570}$:DSG-1$^{378\text{-}500}$	5.0×10^{-08}	32	20,000	640,000
SdrD$^{244\text{-}1129}$:DSG-1$^{378\text{-}500}$	2.5×10^{-04}	3	700	2,100
B-domains effect	2.5×10^{-04}	4	17	68
**Total p_{PROX}**				754,168
**Total all**				760,168

Data S1. A folder containing all datasets used to generate main and supplementary figures.
